# Evolutionary preservation of CpG dinucleotides in RAG1 may elucidate the relatively high rate of methylation-mediated mutagenesis of RAG1 transposase

**DOI:** 10.1007/s12026-023-09451-8

**Published:** 2024-01-19

**Authors:** Mariam M. Fawzy, Maiiada H. Nazmy, Azza A. K. El-Sheikh, Moustafa Fathy

**Affiliations:** 1 Department of Biochemistry, Faculty of Pharmacy, Minia University, Minia, 61519 Egypt; 2 Basic Health Sciences Department, College of Medicine, Princess Nourah bint Abdulrahman University, 11671 Riyadh, Saudi Arabia

**Keywords:** CpG methylation, RAG, V(D)J recombination, Transposition, Mutagenesis

## Abstract

Recombination-activating gene 1 (RAG1) is a vital player in V(D)J recombination, a fundamental process in primary B cell and T cell receptor diversification of the adaptive immune system. Current vertebrate RAG evolved from RAG transposon; however, it has been modified to play a crucial role in the adaptive system instead of being irreversibly silenced by CpG methylation. By interrogating a range of publicly available datasets, the current study investigated whether RAG1 has retained a disproportionate level of its original CpG dinucleotides compared to other genes, thereby rendering it more exposed to methylation-mediated mutation. Here, we show that 57.57% of RAG1 pathogenic mutations and 51.6% of RAG1 disease-causing mutations were associated with CpG methylation, a percentage that was significantly higher than that of its RAG2 cofactor alongside the whole genome. The CpG scores and densities for all RAG ancestors suggested that RAG transposon was CpG denser. The percentage of the ancestral CpG of RAG1 and RAG2 were 6% and 4.2%, respectively, with no preference towards CG containing codons. Furthermore, CpG loci of RAG1 in sperms were significantly higher methylated than that of RAG2. In conclusion, RAG1 has been exposed to CpG mediated methylation mutagenesis more than RAG2 and the whole genome, presumably due to its late entry to the genome later with an initially higher CpG content.

**Supplementary information:**

The online version contains supplementary material available at 10.1007/s12026-023-09451-8.

## Introduction

Significant numbers of primary immunodeficiencies, such as severe combined immunodeficiency (SCID) and Omenn syndrome (OS), result from mutations in the recombination-activating gene (*RAG*). RAG1 and RAG2 proteins, encoded by the *RAG1* and *RAG2* genes, are critical for V(D)J recombination process to recombine V (Variable), D (Diversity), and J (Joining) gene segments at antigen receptor loci and in turn, generate a vast array of the productive immunoglobulin, or T cell receptor exons during lymphocyte development. The V, D, or J gene segments are abutted by DNA recombination signal sequences (RSSs) and are specifically recognized by RAG1 [1]. RAG1 (within the RAG1:RAG2 complex) binds to the RSSs to induce double DNA breaks (DDB) next to the coding segments to generate coding ends and joint ends. Both ends are processed by a non-homologous end joining (NHEJ) pathway [2]. RAG1 is proposed to have evolved from a RAG transposon that entered the vertebrate genome through horizontal gene transfer 500 million years ago [3] and underwent a domestication process to generate RAG recombinase with diminished transposition activity to perform the highly specialized function for a powerful adaptive immune system [4]. Being a former transposon, RAG1 is predicted to have been exposed to methylation during evolution as one main role of DNA methylation is inducing transcriptional silencing of transposable elements (TEs) that pose a continuous threat to the genome stability due to its intrinsic mobile nature [5–7].

DNA methylation occurs predominately, but not exclusively, in the CpG dinucleotides, where a methyl group is introduced in the 5-carbon of Cytosine, followed by spontaneous hydrolytic deamination reaction converting cytosine into thymine (T) [8]. Cytosine (C) and guanine (G) bases account for 40% of the human genome. Although the hypothetical expected content for CpG dinucleotides is 0.04, the actual value is between 0.008 and 0.01 [1–3]. This discrepancy is chiefly mediated by DNA methylation-mediated mutagenesis. Indeed, 70–80% of CpG dinucleotides in the human genome are 5-methylated [9].

This study investigated to what extent methylation-induced mutagenesis has contributed to *RAG1* disease-causing mutations by exploring online resources and checking the inheritance of methylation-mediated mutations in *RAG1* compared to *RAG2*. Furthermore, it aimed to test the hypothesis that the high mutation rate of *RAG1* is because it still has many of its original CpG dinucleotides and, thus, is more prone to methylation-mediated mutagenesis compared to other genes. This purpose was achieved by analyzing the CpG densities and scores in the ancestral genes of *RAG1* and checking the ancestral CpG in RAG1/RAG2 coding sequences of other vertebrates. Finally, the study checked if the relatively high CpG density of *RAG1* caused preference towards CG-containing codons when compared to *RAG2* and the whole genome.

## Materials and methods

### Software

The following software were used in the current study: MegaX (Molecular Evolutionary Genetics Analysis) [10] 64-bit, Excel, Expasy translate online tool.

### Mining and investigation of RAG1 and RAG2 mutations in publicly available data

#### Online repositories

The present study was concerned with substitutional point mutations identified through the coding sequences of *RAG1* and *RAG2* in the National Center for Biotechnology Information (NCBI) section of (ClinVar). The analysis excluded frameshift mutations. Mutations caused by CpG methylation (these were converted only to TpG or CpA) were identified by manual mapping of each mutation on *RAG1* and *RAG2* coding sequences downloaded from ensemble and aligned with its protein translate (ExPASy – Online Translate tool) to confirm mutation position and codon change. The clinical significance of mutations in NCBI was as pathogenic(P), likely pathogenic (LP), benign (B), likely benign (LB), uncertain significance (Un.S), and conflicting interpretation of pathogenicity (CP). The website uses the term ‘conflicting interpretation of pathogenicity’ (CP) to describe mutations having conflicting data from different submitters. Our analysis included pathogenic, likely pathogenic mutations and ‘CP’ mutations submitted as P or LP more times than other interpretations. Supplementary tables S1 and S2 for *RAG1* and *RAG2* contain codon change, methylation status and clinical significance for each mutation. Finally, the percentage of (CpG) methylation-mediated mutations among all mutations linked to pathogenicity was calculated for *RAG1* and *RAG2*.

#### Published clinical data

The current study examined scientific journals for published clinical data of patients with RAG mutations [11–80] to explore the incidence of CpG methylation-mediated mutations. This examination included patients’ ethnicity that duplicated mutations in patients of the same population were counted as one mutation unless patients belonged to unrelated families. Furthermore, we analyzed the data displayed by Lawless et al. [49] who innovated a tool referred to as the average mutation rate residue frequency (MRF) to predict the likelihood of clinically related mutations in *RAG1* and *RAG2*. The higher the MRF score was, the higher the possibility of occurrence of clinically related mutations. They displayed MRF for a list of *RAG1* and *RAG*2 mutations. The current study categorized mutations into CpG and non-CpG methylation-induced mutations and calculated the average MRF and *p*-value.

### Investigation of the methylation ratio of CpG loci in RAG1 and RAG2 by using the online available bisulfite seq data analysis of spermatozoa cells

To check if the methylation-mediated mutations are inherited, this study examined the methylation in the germ cell (sperm). The current study determined methylated CpG loci of RAG1 and RAG2 by using the online available bisulfite sequence analysis, which depends on using bisulfite before the high-throughput sequencing for the differentiation between methylated and non-methylated cytosine. Bisulfite converts the non-methylated cytosine to uracil, which then is read as thymine through sequencing, while methylated cytosine remains unchanged [81].

The genome data viewer displayed the methylation pattern of only one sample via the link https://www.ncbi.nlm.nih.gov/geo/query/acc.cgi?acc=GSM1127119, and UCSC (University of California Santa Cruz) epigenome viewer Human chr11:36589563-36601310 - UCSC Genome Browser v309 (epigenomebrowser.org). For sampling, testis spermatozoa primary cells Donor 390ATA mapped by Illumina Bisulfite-Seq read and mappings were processed into graphs of methylation proportions. The genome viewer included the whole genome of the sample with an option to zoom in on the required position. The present study interpreted the methylation percent of the CpG loci from the genome browser, and calculated the percentage of each category, mean, and *p*-value between methylation ratios in RAG1 and RAG2.

### Estimation of CpG density and CpG score in the ancestral RAG genes

As the proposed ancestors for RAGs, the sequences of the potential RAG relative: *Hztransib*, *spRAG1L*, *spRAG2L*, and *ProtoRAG* were downloaded from NCBI, and their CpG densities were estimated. The CpG density was identified by calculating the total number of CG dinucleotides in the gene and comparing this to the overall gene length using the (Len) function in Microsoft Excel. CpG density was expressed as a percentage.

CpG score is a known tool used to assess the extent of genome exposure to DNA methylation [82, 83]. The higher the CpG score, the lower exposure to DNA methylation is. The CpG score was calculated by dividing the observed CpG density by the expected CpG density (G+C)/2)2.

### Examination of ancestral CpGs for RAG1 and RAG2

The *RAG1* cDNAs from different species were aligned and used to estimate the number of CpGs in the ancestral gene. The “Fasta” files for *RAG1/RAG2* coding sequences of 40 species were downloaded from the Ensemble. Species were selected to be representative of all categories: birds, reptiles, rodents, primates, mammals, and fish. The alignment results were exported from MegaX to Excel and then printed. CpG dinucleotides were checked throughout all 40 species, and we marked the ones where it is likely to have mutated via methylation-mediated mutagenesis. If there was just one or two CpGs at a position in all 40 species, this was likely to have arisen rather than to have been mutated in the remaining 39 species. Therefore, the ancestral CpG dinucleotides were marked when detected in 3 or more species with seven or more TG/CA dinucleotides in other species or when CpG dinucleotides were detected in 10 or more species in a position. Finally, the percentage of these columns to the total number of nucleotides in the coding sequence was estimated and represented the ancestral CpG density.

### Analysis of CG containing codons in RAG1 and RAG2 compared with that in the human genome

RAG1 is a DNA-binding protein, so it is expected to have high Arginine “R” residues. Six codons encode Arginine: four CGXs (X is G, C, A or T), AGA, and AGG. We aimed to investigate whether most arginine residues are encoded by CGX, which may be the reason for RAG1’s high CpG density. The sequence analysis website (Codon Usage Calculator - Free Online Analysis Tool - BiologicsCorp) verified the fraction of each arginine codon in RAG1.and results were compared with that in RAG2 and the human genome. Additionally, any other CG-containing codons were checked in both RAG1 and RAG2. Generally, CG-containing codons are CGT, CGC, CGA, and CGG for arginine; GCG for alanine; TCG for serine; CCG for proline; and ACG for threonine amino acids. These codons were also checked in the human genome using another sequence analysis tool called GenScript Codon Usage Frequency Table (chart) (https://www.genscript.com/tools/codon-frequency-table).

### Statistical analysis

An independent sample *t*-test was used to compare the means between the two groups. The *Z*-score test was used to compare between two proportions.

## Results

### Pathogenic RAG1 mutations were associated with high CpG methylation status

**Fig. 1:**
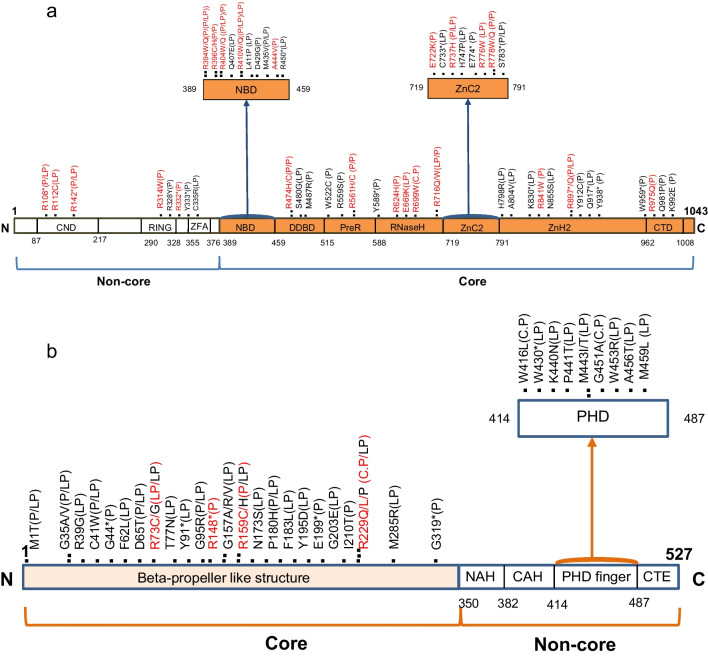
Point mutations with pathogenicity of RAG1 and RAG2 proteins. **a** shows 59-point mutations with pathogenicity over the RAG1 protein main domains, 50.8% of them are methylation-mediated mutations marked with red font. (noncore domains: CND, central non-core domain; RING, really interesting new gene, ZFA, zinc finger A, Core domains: NBD, nonamer binding domain; DDBD, dimerization and DNA binding domain; PreR, Pre-RNAse H; CTD, C-terminal domain); **b** shows 41-point mutations with pathogenicity spreading over the RAG2 protein main domains, 12.19% of them are methylation-mediated mutations. (non-core domains: NAH; N-terminal acidic hinge, CAH, C-terminal acidic hinge, CTE; C-terminal extension)

RAG1 and RAG2 mutations are reportedly frequent in multiple primary immunodeficiencies. Mining the ClinVar tool at the NCBI database identified 393-point mutations at the coding sequence of RAG1, from whom 59 mutations were linked to disease pathogenicity. Stratifying pathogenic *RAG1* mutations revealed that 33/59 (55.9%) mutations were purely pathogenic, 17/59 (28.8%) were likely pathogenic, 8/59 (13.56%) were reported to be pathogenic or likely pathogenic, and only 1/59 (1.69%) mutation conflicting with pathogenicity (Fig. 1a). To investigate if RAG1 mutations with their related pathogenicity might be caused by CpG methylation, mapping the *RAG1* Open Reading Frame (ORF) for CpG methylation was manually performed after excluding mutations marked with mixed or conflicting pathogenicity. 57.57% (19/33) of the purely pathogenic *RAG1* mutations had at least one C: G > T: G or C: G > C: A change compared to 23.5% (4/17) mutations assigned as likely pathogenic (*z* score = 2.2882, *p*-value = 0.02) suggesting higher frequency of CpG methylation in *RAG1* mutations that had clear pathogenic role across different disease etiologies.

The coding sequence of RAG2 had 216-point mutations, 41 of which were described to have pathogenicity. Again, stratifying pathogenic RAG2 mutations revealed that 7/41 (17.07%) mutations were purely pathogenic, 26/41 (63.4%) were likely pathogenic, 5/41 (12.19%) were reported to be pathogenic or likely pathogenic, and 3/38 (7.89%) mutation conflicting with pathogenicity (Fig. 1b). Unlike RAG1, only 28.57% (2/7) and 7.69% (2/26) of the pure and likely pathogenic *RAG2* mutations, respectively, were linked to CpG methylation (*z* score = −1.5, *p*-value = 0.13) probably highlighting other causative factors that are linked with RAG2 pathogenicity. As expected, the percentage of CpG methylation-mediated pathogenic mutations in *RAG1* was significantly higher than that of *RAG2* (50.8% versus 12.19%, *z* score = 3.9857, *p*-value < 0.0001).

To validate this data, we investigated papers reporting the role of RAG1 and RAG2 in primary immunodeficiencies and their methylation status. In line with the NCBI data, 51.64% (110 out of 213) of the total number of mutations in cases with immunodeficiencies or autoimmunity had CpG mutations mediated by methylation in *RAG1* coding sequence compared to 28.86% (28/97) in *RAG2* coding sequence (*z* score = 3.74, *p* value = 0.0002). Noteworthy, the CpG mutations mediated by methylation in the human genome were about 31.5%, a percentage lower than CpG mutations mediated by methylation in the *RAG1* coding sequence (*z* score = 3.25, *p* value = 0.001).

To investigate the probability of mutation for each amino acid residue in RAG1 and RAG2 proteins, Lawless and his colleagues innovated the average mutation rate residue frequency (MRF) tool by multiplying residue frequency by mutation rate per residue [49]. They found a positive correlation between MRF and the prediction of *RAG1*, but not *RAG2*, mutation-related pathogenicity. In 66 pathogenic *RAG1* mutations analyzed by the same research group, we identified 27 CpG methylation-mediated mutations and 39 non-CpG mutations with an average MRF of 0.04 and 0.02, respectively (*t*-value = 7.41515, *p* < 0.0001). 23/27 CpG-related *RAG1* mutations (85.18%) had an MRF maximum value (MRFmax) of 0.043 compared to the 0/41 *RAG2* mutation. In conclusion, the high frequency of CpG mutations mediated by methylation in the *RAG1* (but not in the whole genome or RAG2) coding sequence suggests a role of DNA methylation in the pathogenesis of RAG1 mutation.

To discern whether high CpG frequency in the *RAG1* coding sequence is inherited or acquired, *RAG1* methylation status in sperm samples should be checked. Typically, methylation needs to occur in the germ cells/progenitor cells for the mutations to happen and is manifested subsequently in lymphocytes. Searching the Genome Expression Omnibus (GEO) database for sperm bisulfite sequencing identified a study that includes one sample (donor 390ATA, accession: GSM1127119). Analyzing *RAG1* and *RAG2* methylation status in that study showed that around 90.19% of *RAG1* and 55.56% of *RAG2* CpG loci had a high methylation level. The methylation ratio of CpG loci in the RAG1 sequence was significantly higher than that in RAG2 (*p*-value = 0.001), suggesting that the high methylation ratio in the *RAG1* sequence compared to *RAG2* might be inherited.

### The CpG dinucleotides frequency was higher in the ancestral RAG transposons

*RAG1* evolved from RAG transposon and was introduced into the vertebrate genome half a billion years ago. Investigation of the methylation status in *RAG* ancestors might explain the higher CpG density shown in human *RAG1*. *Transib*, *SpRAG1L* and *SpRAG2L* (in sea Urchin), and the *ProtoRAGs BbRAG1L* and *BbRAG2L* (in Lancelet) are DNA transposal superfamilies that had a sequence similarity to human *RAG*. As in Table 1, the CpG density (and CpG score) for *Helicoverpa Zea Transib*, *SpRAG1L*, *SpRAG2L*, *BbRAG1L*, and *BbRAG2L* were 3% (0.83), 3.27% (0.48), 2.5% (0.5), 2.7% (0.48), and 2.7% (0.49), respectively, compared to 1.6% (0.27) and 0.56% (0.12) in human *RAG1* and *RAG2*. High CpG densities and scores for all *RAG* relatives in comparison with the current *RAG* suggest that the original *RAG* transposons, from which the current *RAG* evolved, had high CpG density and were exposed to mutagenic deamination until they reached the current CpG density of 1.6% in *RAG1*.

**Table 1:** The number and percentage of CpG and CpG score in the ancestral RAGs

Name	Number of CpG	CpG density	CpG score (CpG o/e ratio)
*Helicoverpa* *Zea* *Transib*	44	3%	0.83
*(Sea Urchin) SpRAG1L*	98	3.27%	0.48
*SpRAG2L*	38	2.5%	0.5
*Lancelet ProtoRAG*	128	2.7%	0.48
*BbRAG1L*	93	2.7%	0.49
*BbRAG2L*	35	3.14%	0.48
*Homo Sapiens RAG 1*	51	1.6%	0.27
*Homo Sapiens RAG 2*	9	0.56%	0.12

**Fig. 2:**
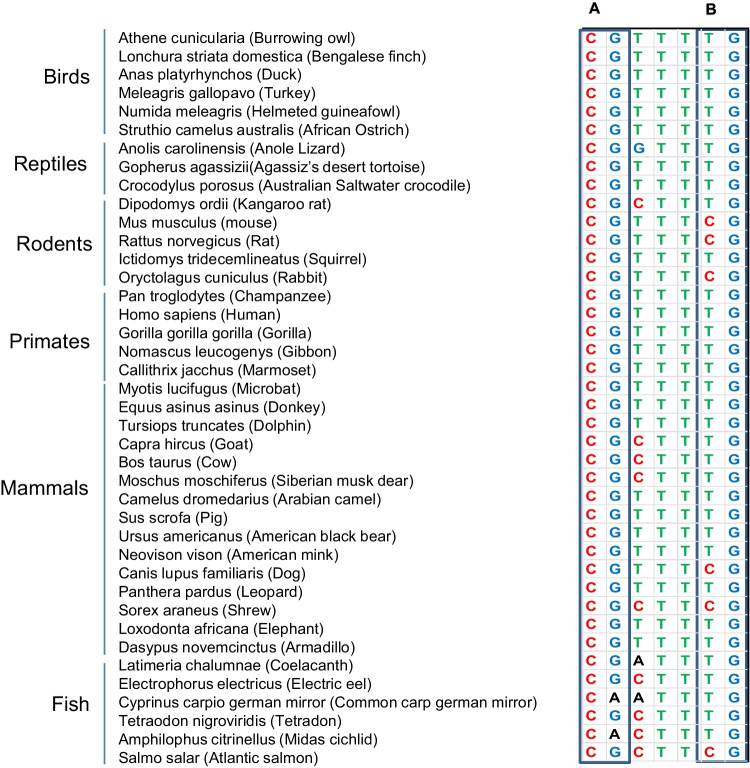
The representative figure for alignment of *RAG1* coding sequences from 40 different species categorized into birds, reptiles, rodents, primates, mammals, and fish. Columns A, B, and C represent columns with ancestral CpGs through the species. Column A has 5 CG and 27 CA, and column B has 6 CG and 34 TG

**Fig. 3:**
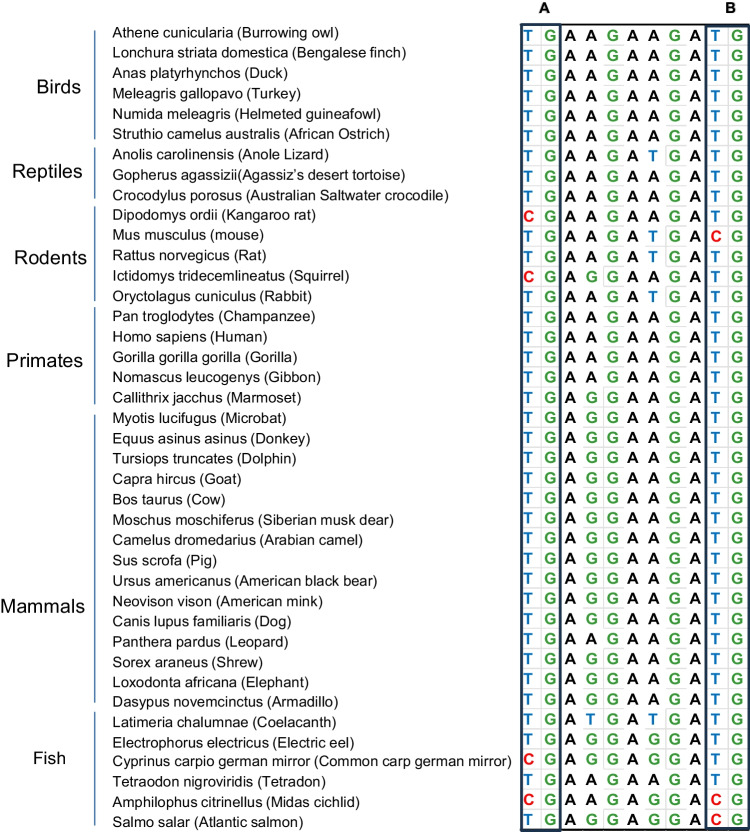
The representative figure for alignment of *RAG2* coding sequences from 40 different species categorized into birds, reptiles, rodents, primates, mammals, and fish. Columns A, B, and C represent columns with ancestral CpGs through the species. Column A has 4 CG and 36 TG, and column B has 3 CG and 37 TG

After assessing the DNA methylation frequency in *RAG* ancestors, the study performed a subsequent comparative analysis of the CpG frequency in *RAG1* and *RAG2* sequences in other vertebrates. One hundred eighty-nine conserved/mutant CG dinucleotides were identified across the aligned *RAG1* coding sequences in 40 species, 23 of whom were conserved. CG > TG and CG > CA nucleotide changes were observed 95 and 56 times, respectively, while 15 CG > CA or TG loci were detected (Fig. 2). The vertebral CpG density in the *RAG1* coding sequence, denoted by the total number of CpG loci (189) divided by the 3132 nucleotides in the *RAG1* coding sequence, was 6.03% compared to only 4.2% (67 out of 1584) in the *RAG2* coding sequence (*z* score = 2.583, *p* value = 0.00988) (Fig. 3; supplementary table S3) further confirming the higher abundance of CpG in vertebral *RAG1* sequence.

### High abundance of CpG in RAG1 did not confer preference towards CG-containing codons

RAG1 directly binds and cleaves DNA at the border of signal sequences, while RAG2 does not have a DNA binding affinity but instead forms a RAG1-RAG2 complex. One explanation of the RAG1 DNA binding activity is the higher Arginine amino acid content compared to that in RAG2. Arginine can be encoded by six codons (four CGX codons, AGA, or AGG), so the relatively large numbers of CpG dinucleotides in RAG1 may induce a preference towards CGX codons encoding arginine. To test this hypothesis, arginine-encoding sequences in *RAG1* and *RAG2* were counted. Table 2 shows that the RAG1 protein includes 66 arginine residues: 31 CGX codons (46%) and 36 AGA/AGG codons (54%), whereas the human genome contains 60% CGX and 40% AGA/AGG (GenScript website). Other amino acids encoded by XCG codons, including alanine, serine, proline, and threonine, were also analyzed for their CG content. Once more, only 2/68 of alanine-encoded codons were GCG (2.9%), 2/85 serine-encoded codons were TCG (2.3%), 2/52 proline-encoded codons were CCG (3.8%), while 2/40 (5%) threonine-encoded codons were ACG. Interestingly, the prevalence of these amino acids CG-containing-codons was lower than the corresponding human genome. Regarding *RAG2*, 8/18 of arginine-encoding codons were CGX (44.45%), 10/18 were AGA/AGG (55.55%), while alanine, serine, proline, and threonine amino acids were neither encoded by GCG, TCG, CCG, nor ACG in RAG2 protein. In conclusion, high CpG content in the *RAG1* coding sequence did not confer any codon-usage bias in the relevant protein.

**Table 2:** The percentage of CG containing codons (with red font) compared to non-CG-containing codons for amino acids: arginine, alanine, serine, proline, and threonine in RAG1, RAG2, and the human genome

Amino acid codons		RAG1			RAG2			Human genome		
		Percentage		Number	Percentage		Number	Percentage		Number
Arginine	CGT	12.12	45.5	8	11.11	44.45	2	8	60	93,458
	CGC	6		4	22.22		4	19		217,130
	CGA	6		4	5.5		1	11		126,113
	CGG	21.2		14	5.5		1	21%		235,938
	AGA	19.7	54.5	13	44.44	55.55	8	20	40	228,151
	AGG	34.8		23	11.11		2	20		227,281
Alanine	GCT	35.3		24	30		6	26		370,873
	GCC	35.3		24	40		8	40		567,930
	GCA	26.5		18	30		6	23		317,338
	GCG	2.9		2	Zero		Zero	11		150,708
Serine	TCT	26.4		23	31		13	18		291,040
	TCC	23		20	16.67		7	22		346,943
	TCA	15		13	21.4		9	15		233,110
	TCG	2.3		2	Zero		Zero	6		89,429
	AGT	17.24		15	16.67		7	15		237,404
	AGC	16		14	14.29		6	24		385,113
Proline	CCT	32.7		17	31		9	28		343,793
	CCC	15.4		8	27.6		8	33		397,790
	CCA	48		25	41.4		12	27		331,944
	CCG	3.8		2	Zero		Zero	11		139,414
Threonine	ACT	37.5		15	37		13	24		255,582
	ACC	43.5		17	17.14		6	36		382,050
	ACA	15		6	45.7		16	28		294,223
	ACG	5		2	Zero		Zero	12		123,533

## Discussion

RAG-mediated V(D)J recombination is essential for durable adaptive immunity. Therefore, mutations in the human RAG genes are correlated with a significant number of immunodeficiencies. In this study, we tried to determine whether the high mutation rate of *RAG1* was because it retained many of its original CpG and, consequently, was more exposed to methylation-mediated mutagenesis than other genes. This study is the first to check the extent of CpG methylation contribution in RAG disease-causing mutations. A review of NCBI-identified pathogenic mutations in the coding sequences of *RAG1* demonstrated that CpG methylation was the causative for 57.57% of these mutations. Even after including others described as likely pathogenic and conflicting interpretations with pathogenicity (specifically that has been submitted as pathogenic/likely pathogenic more often than other clinical significance interpretations), the percentage remained high (50.8%) and was significantly higher than 12.19%, the percent of *RAG2* point mutations caused by CpG methylation to the whole RAG2 mutations linked to pathogenicity (*p*-value < 0.0001).

Then, this study inspected published clinical cases with immunodeficiencies and found that 51.6% and 28.86% of patients with *RAG1* and *RAG2* mutations, correspondingly, had CpG methylation-mediated mutagenesis (*p*-value=0.0002). Additionally, the RAG1 percentage is significantly higher than 31.5%, the percentage of the disease-causing methylation-mediated mutations in the human genome (*p*-value = 0.001) [84]. These findings agreed with the MRF values calculated by Lawless et al. [49] and analyzed in the present work. They used population genetics data from about 146,000 individuals for minor variant analysis. To validate the calculated scores of MRF, they used 44 previously identified pathogenic variants stated in patients and recombination activity scores from 110 mutated RAG1/2. Likewise, they compared probabilities with 98 currently reported diseased cases in humans. They also used a genome sequence dataset of 558 patients with primary immunodeficiency/wild-type RAG as negative controls. They found a positive correlation between the MFR values and pathogenicity prediction of RAG1 and not RAG2 mutations [49].

Although CpG methylation-mediated mutation is known to be inherited [85], we had to confirm the methylation status at the CpG loci in RAG1 and RAG2 in specific way. After examining the methylation level through the online available bisulfite seq analysis of one spermatozoa sample, methylation levels for the CpG loci observed in *RAG1* were significantly higher than in *RAG2* (*p*-value = 0.001), which might explain the higher mutation levels in *RAG1* than in *RAG2* in agreement with Zhou et al. who found a positive correlation between the methylation level and the mutational rate in the human germline (sperms and oocytes) when analyzed by whole genome bisulfite sequencing during the development stage [86].

Following that, the study undertook to identify the reason for the relatively high incidence of CpG methylation-mediated mutations in *RAG1* by examining its evolutionary roots. Since identifying V(D)J recombination, the standard features between this process and cut-and-paste transposition have had significant attention [87]. RAG cleaves adjacent to RSS, which is reminiscent of inverted terminal repeats (TIRs) targeted by transposases, but instead of NHEJ, the transposon is inserted into the target DNA generating characteristic edges called target site duplication (TSD), whose length is variable according to the TE. These features, along with the discovery of RAG-mediated transposition, strengthen the hypothesis of V(D)J recombination evolution known as transposon/split receptor gene, which assumed that *RAG1*, *RAG2*, and the gene segments of antigen receptor loci have originated from the TE containing *RAG1*-like (*RAG1L*) and *RAG2L* genes flanked with TIRs [87–89]. Vertebrates’ RAG emerged by horizontal transfer to the genome of jawed vertebrates as a RAG transposon at the time of the emanation of their complex adaptive immune system [90–92]. Believed all adaptive immunity components arose about 500 million years ago after the division of jawless vertebrates without any known source in the ancient species. So, it was called the immunological “big bang” [93]. Sequence similarities were identified between the current *RAG1* and *Transib* family of TEs. *RAG1* consists of an active core region and a regulatory non-core region (Fig. 1). *Transib* resembles only the core region; its TIRs are like RSS, especially the heptamer. *Hztransib* is the active member of this family and has *a RAG*-like transposition manner, including the five bp TSDs left after transposition, so it is considered a precursor for RAG transposon [91]. However, the *Transib* family lacks *RAG2L*. Both *RAG1L* and *RAG2L* are in the purple sea urchin (*Strongylocentrotus purpuratus (SP)*), which has an established evolution relation with the human genome [94]. *SPRAG1L* is like core *RAG1* in addition to the N-terminal RING domain of the non-core, and *SPRAG2L* is like *RAG2*. Unlike other transposons, they have no TIRs or TSDs [92].

The *ProtoRAG* from the Chinese lancelets (*Branchistoma belcheri (Bb*)) consists of *BbRAG1L* and *BbRAG2L* genes, which have structural similarities with *RAG1* and *RAG2*, respectively. Additionally, they are convergently transcribed (as in the case of *RAG1* and *RAG2*) and flanked with RSS-like TIRs. They left five bp TSDs after transposition. The structural similarity between *BbRAG1L* and *RAG1* exceeds the core and extends to include the RING/Zinc finger in the non-core region’s N-terminal region [92]. Many studies suggested models to illustrate how *RAG* has evolved from these transposons [4, 95–97], starting with Hztransib as the most ancient ancestor, passing with modifications and acquisition of *RAG2L* to get *SPRAGL* or *ProtoRAG*, which underwent further changes to diminish the transposition activity and yield the current sophisticated immune system. The process of *RAG* transposon adaptation over the years, giving rise to the existing RAG, is called Transposon domestication [98]. The methylation status of the ancestral RAGs was checked by calculating their CpG densities and scores. Zhou and his colleagues suggested the CpG score as an indicator of the rate of germline CpG mutations through evolution. Additionally, they found an inverse correlation between the age of TEs and CpG density, in turn, CpG score [99].

Table 1 shows that CpG densities and scores for all RAG relatives were higher than that of the human RAG1, confirming that the original RAG1 transposon had higher CpG density when entered our genome. It is worth mentioning that CpG scores for *SpRAG2L* (0.5) and *BbRAG2L* (0.49) are slightly higher than that for *SpRAG1L* (0.48) and *BbRAG2L* (0.48), which is quite different from the present case of the human *RAG* where *RAG2* CpG score (0.12) is lower than half that of *RAG1*(0.27) and even lower than that of the whole genome (0.2-0.25). This lower-than-expected CpG score of *RAG2* is against the assumption that RAG2 entered the genome later than RAG1 and the hypothesis of Kapitonov and Koonin about the common transposon from which both proteins evolved [3] unless *RAG2* was exposed exclusively to extensive CpG methylation mediated mutagenesis during evolution.

Besides, the CpG conversion into CpA, TpG, or both was identified in the aligned 40 RAG coding sequences from different vertebrates to investigate the original number of CpG dinucleotides in the ancient vertebrate *RAG*. *RAG1* had a higher fraction of ancestral (CpG)s (6%) than *RAG2* (4.2%) and the current *RAG1* CpG fraction (1.6%). Remarkably, we found the expected CpG content for *RAG1* and *RAG2* to be 6% and 4.5%, respectively, based on the (C + G) percent in *RAG1* (48.7%) and *RAG2* (42.5%).

Lastly, the study tried to check if there is a relation between the comparatively high CpG density in RAG1 and the abundance of CG-containing codons. Non-even usage for the synonymous codons is observed in different species and is known as codon usage bias (CUB) [100]. Preference to specific codons rather than others of the same amino acid is affected mainly by mutation and natural selection [101, 102]. Checking the CG-containing codons has revealed that the relatively high CpG density in *RAG1*, compared to *RAG2* and the whole genome, was not related to the high arginine residues present in such a DNA-binding protein like *RAG1*, as 54% of arginine residues were encoded with AGA/AGG, while the other four codons (CGG, CGC, CGA, and CGT) encoded only 46%. However, most arginine codons (60%) in the human genome were CGX, while only 40% were AGA/AGG.

## Conclusions

Disease causing methylation-mediated mutations occurred more frequently in RAG1 coding sequence compared to *RAG2* and the human genome. *RAG1* had higher CpG density and CpG score than *RAG2* and the human genome, so it seemed that *RAG1* kept most of its original CpG dinucleotides. Further research should be done to discover the exact mechanism behind the extremely high methylation rate experienced by *RAG2* during evolution.

### Supplementary information

Below is the link to the electronic supplementary material.

Supplementary file1 (PDF 1397 KB) The following supporting information can be downloaded at https://www.mdpi.com/xxx/s1

## Data Availability

Data generated or analyzed during this study are provided in full within the published article and its supplementary materials.
