# Supplementary material for: Evolutionary preservation of CpG dinucleotides in RAG1 may elucidate the relatively high rate of methylation-mediated mutagenesis of RAG1 transposase
Source: Immunol Res. 2024 Jan 19;72(3):438–49. doi: 10.1007/s12026-023-09451-8 (PMC11217092; doi:10.1007/s12026-023-09451-8)
Supplement: Supplementary file 1 — Supplementary file1 (PDF 1397 KB) The following supporting information can be downloaded at https://www.mdpi.com/xxx/s1 [file 12026_2023_9451_MOESM1_ESM.pdf]

TableS1: List of mutations in RAG1 coding sequence:

No		Mutation	Codon change		Clinical# Significance	Phenotype	MRF ¥ [1]	Citation	no
1.	#	R34Q	CGG-CAG	+	Uncertain Significance (Un.S)	Combined cellular and humoral immune defects with granuloma			
2.	#	R34L	CGG-CTG		Un.S	Combined cellular and humoral immune defects with granuloma			
3.	#	p. His92=	CAC GAC -CAT GAC	+	C.P (Conflicting interpretation with pathogenicity) (2 Un.S, 2 LB)	Not provided (NP)			
4.	#	H92Q	CAC GAC-CAA GAC		Un.S	Severe Combined Immune Deficiency (SCID), T^-, B^-, NK^+			
5.	#	D93N	CAC GAC-CAC AAC		Un.S	Combined cellular and humoral immune defects with granuloma			
6.	#	E95K	AAC GAG-AAC AAG	+	Un.S	Combined cellular and humoral immune defects with granuloma			
7.	#	p. Ala101=	GCG-GCA	+	Benign(B)	SCID, not specified, Histiocytic medullary reticulosis (HMR), , Combined cellular and humoral immune defects with granulomas			
8.	#-	R108*	CGA-TGA	+	Pathogenic(P)/Likely Pathogenic (LP)	Atypical SCID Combined cellular and humoral immune defects with granuloma	0.043	1 patient homo[2] 2 unrelated Chinese hetero[3, 4] 1Belarus patient hetero[5]	4
9.	#	R108Q	CGA-CAA	+	Un.S	SCID, T^-, B^-, NK^+			

10.	#-	R112H	CGC-CAC	+	Un.S	Immunodeficiency, common variable & lymphoma Combined cellular and humoral immune defects with granuloma			
11.	#	R112C	CGC-TGC	+	LP	Inherited immunodeficiency # diseases			
12.	#-	R112L	CGC-CTC		Un.S	SCID			
13.	#-	R142*	CGA-TGA	+	P/LP	Omenn syndrome Combined cellular and humoral immune defects with granulomas, Alpha/beta T-cell lymphopenia with gamma/delta T-cell expansion, severe cytomegalovirus (CMV)infection, and autoimmunity, HMR,	0.043	1 Japanese hetero[6]<br>1 Italian patient hetero[2, 7]<br>1 Egyptian hetero[8]<br>1 pt hetero with unknown [2]<br>1 from Russia [9]	4
14.	#-	R142Q	CGA-CAA	+	Un.S	SCID		1 Turkish hetero[10]	1
15.	#	p. Pro152=	CCG-CCA	+	Un.S	Combined immunodeficiency with skin granulomas, Severe combined immunodeficiency, autosomal recessive, T cell-negative, B cell-negative, NK cell-positive			
16.	#	P152L	CCG-CTG	+	Un.S	Combined immunodeficiency with skin granulomas, Severe combined immunodeficiency, autosomal recessive, T cell-negative, B cell-negative, NK cell-positive			
17.	#	S169L	TCG-TTG	+	LB	Combined immunodeficiency with skin granulomas, Severe combined immunodeficiency, autosomal recessive, T cell-			

						negative, B cell-negative, NK cell-positive			
18.	#	S169W	TCG-TGG		Un.S	NP			
19.	#	p. Pro197=	CCG-CCA	+	LB	Combined cellular and humoral immune defects with granulomas, HMR, Alpha/beta T-cell lymphopenia with gamma/delta T-cell expansion, severe cytomegalovirus infection, and autoimmunity,			
20.	#	Asn199=	AAC GTG-AAT GTG	+	C.P<br>(2 Un.S,1 LB)	Severe combined immunodeficiency, autosomal recessive, T cell-negative, B cell-negative, NK cell-positive, HMR, Combined immunodeficiency with skin granulomas, SCID, autosomal recessive, T cell-negative, B cell-negative, NK cell-positive			
21.	#	V200M	AAC GTG-AAT GTG		Un.S	Combined immunodeficiency with skin granulomas, Severe combined immunodeficiency, autosomal recessive, T cell-negative, B cell-negative, NK cell-positive			
22.	#	R218H	CGT-CAT	+	Un.S	HMR, SCID			
23.	#	R218C	CGT-TGT	+	Un.S	SCID, autosomal recessive, T-cell negative, B-cell negative, NK-cell positive, combined immunodeficiency with skin granuloma			
24.	#	R219W	CGG-TGG	+	Un.S	SCID, autosomal recessive, T-cell negative, B-cell negative, NK-cell positive, combined			

						immunodeficiency with skin granuloma			
25.	#	R219Q	CGG-CAG	+	Un.S	Combined cellular and humoral immune defects with granuloma			
26.	#	R219L	CGG-CTG		Un.S	Combined cellular and humoral immune defects with granuloma			
27.	#	R247G	CGT-GGT		Un.S	SCID, autosomal recessive, T-cell negative, B-cell negative, NK-cell positive, combined immunodeficiency with skin granuloma			
28.	#	R247C	CGT-TGT	+	Un.S	Combined cellular and humoral immune defects with granuloma			
29.	#	R247L	CGT-CTT		Un.S	SCID, autosomal recessive, T-cell negative, B-cell negative, NK-cell positive, combined immunodeficiency with skin granuloma			
30.	#	R247H	CGT-CAT	+	Un.S	SCID, T^-, B^-, NK^+			
31.	#	R249H	CGC-CAC	+	B	SCID, autosomal recessive, T-cell negative, B-cell negative, NK-cell positive, combined immunodeficiency with skin granuloma	0.026	1 Iranian [11]<br>1 Indian Sikh[12]<br>3 unrelated pts Netherland[13]<br>9 pts (3 Arab,6 Jewish) in Israel[14]	7
32.	#	A267T	ATC GCC-ATC ACC	+	Un.S	HMR, Severe Combined Immune Deficiency			
33.	#-	R314W	CGG-TGG	+	P	SCID<br>Combined cellular and humoral immune defects with granuloma		1 German hetero cpd[2, 15, 16]	1
34.	#-	R332*	CGA-TGA	+	P	Omenn syndrome<br>Combined cellular and humoral immune defects with granuloma			
35.	#	R332Q	CGA-CAC	+	Un.S	SCID, autosomal recessive, T-cell negative, B-cell negative, NK-cell positive, combined			

						immunodeficiency with skin granuloma			
36.	#	V350I	AGC GTC-AGC ATC	+	Un.S	Combined cellular and humoral immune defects with granuloma, HMR			
37.	#	V350L	AGC GTC- AGC CTC		Un.S	SCID, autosomal recessive, T-cell negative, B-cell negative, NK-cell positive, combined immunodeficiency with skin granuloma			
38.	#-	R394Q	CGG-CAG	+	P/LP	SCID, T & B cell -ve<br>Combined cellular and humoral immune defects with granuloma	0.043	1 patient homo[17 ,2]	1
39.	#-	R394W	CGG-TGG	+	P	Omenn syndrome<br>Combined cellular and humoral immune defects with granuloma		1 Chinese hetero cpd[3]	1
40.	#-	R396H	CGC-CAC	+	P	Omenn syndrome<br>Severe combined cellular and humoral immune defects with granuloma		1 Japanese who have also R142*[6]<br>1 homo[2]<br>1 Italian hetero [18, 19]	3
41.	#-	R396L	CGC-CTC		NP	Omenn syndrome		1 pt hetero[2, 19]	1
42.	#-	R396C	CGC-TGC	+	P	Omenn syndrome<br>Histiocytic medullary reticulosis	0.043	1 Italian hetero [2, 18, 19]<br>1 American homo [2, 18, 19]<br>1American hetero [2, 20]<br>1 hetro who has R404Q[2]<br>1 in Poland[9]<br>1 in Japan (2 siblings)[6]	5
43.	#	S401L	TCG-TTG	+	Un.S	Combined cellular and humoral immune defects with granuloma			
44.	#	Ser401=	TCG-TCT		LB	SCID, autosomal recessive, T cell-negative, B cell-negative, NK cell-positive, Combined			

						immunodeficiency with skin granulomas			
45.	-	R404Q	CGG-CAG	+	P	SCID	0.043	1 Belarus, Gomal region (who have R108*)[5]<br>2 hetero unrelated pts (one who has R396C[2])	3
46.	#-	R404W	CGG-TGG	+	P/LP	Omenn syndrome<br>Combined cellular and humoral immune defects with granuloma		1 Chinese patient hetero[3]<br>1 French with fs[21]	4
47.	#-	R410Q	CGG-CAG	+	LP<br>(R410Q, A622fs pathogenic)	Atypical SCID/<br>Omenn syndrome	0.043	1 Turkish hetero[22]<br>1 Italian hetero[2, 19]	
48.	-	R410W	CGG-TGG	+	P/LP	Omenn syndrome<br>Combined cellular and humoral immune defects with granuloma		1 hetero[2]	1
49.	#	Ser432=	TCC GTG-TCT GTG	+	LB	Severe combined cellular and humoral immune defects with granulomas, HMR, Alpha/beta T-cell lymphopenia with gamma/delta T-cell expansion, severe cytomegalovirus infection, and autoimmuni			
50.	#-	V433M	TCC GTG-TCC ATG	+	NP	Atypical SCID/<br>Omenn syndrome	0.028	1 hetero[19 ,2]	1
51.	#-	A444V	GCG-GTG	+	P	Atypical SCID/<br>Omenn syndrome	0.027	1 Ukraine, Kiev hetero[5]<br>1 Ukraine, Lisichansk heter[5]<br>1 Turkish hetero[10]<br>1 pt who has V433M[2, 19]<br>1 in Russia<br>2 homo pts[2, 19]<br>Other 2 homo pts[2]	8
52.	-	R474S	CGT-AGT			SCID, T & B cell -ve		1 pt hetero in Italy[23]	1
53.	#-	R474H	CGT-CAT	+	P	Atypical SCID/<br>Omenn syndrome		1 pt heter[19]<br>1 American [24]<br>1 French pt hetero cpd [21]	2

54.	#-	R474C	CGT-TGT	+	P	OS/SCID	0.043	1 German hetero[25]<br>1 Netherlands hetero[2, 26]<br>1 hetero in Utah (2 siblings)[27]<br>1 Japan hetero[2, 28]<br>1 USA hetero[20]	5
55.	#-	R507W	CGG-TGG	+	C.P<br>(1LP, 1Un.S)	SCID, autosomal recessive, T cell-negative, B cell-negative, NK cell-positive, Combined immunodeficiency with skin granulomas			
56.	#	R507Q	CGG-CAG		Un.S	SCID			
57.	#-	R561C	CGC-TGC	+	P<br>(Also R561C, M605I pathogenic)	Omenn syndrome<br><br>SCID	0.043	1 Russian, Samara[5]<br>1 who has R507W<br>1 American hetero [18, 19]<br>1 Asian[29]	4
58.	#-	R561H	CGC-CAC	+	P	OS<br><br>SCID	0.043	1 Turkish pt homo [2, 30]<br>1 French patient homo[2, 21, 30]<br>1 Italian pt homo [2, 18, 30]<br>1 homo[7]	3
59.	#	p. Asp607=	GAC GTG-GAT GTG	+	LB	NP			
60.	#-	R624H	CGT-CAT	+	P<br>(R624H, R405G pathogenic)	SCID, B cell -ve	0.043	1 Ukraine, Kiev[5]<br>1 Chinese hetero[3, 31]<br>1 German hetero<br>1 French hetero[21]	4
61.	#-	R624C	CGT-TGT	+	NP	Omenn syndrome		1 hetero [19]<br>1 American (who has R474H)[24]<br>2 in Serbia[9]	3
62.	#	p. His668=	CAC GAG-CAT GAG	+	B	NP			
63.	#-	E669K	CAC GAG-CAC AAG	+	LP	SCID		1 Chinese hetero[3]	1

64.	#	T670M	ACG-ATG	+	Un.S	Combined cellular and humoral immune defects with granuloma			
65.	-	R699Q	CGG-CAG	+		Immunodeficiency, combined, with granuloma and/or autoimmunity	0.043	1 hetero [2]	1
66.	#-	R699W	CGG-TGG	+	C.P<br>(2 P, 1 L.P, 1 Un.S)	SCID<br>HMR<br>CI with skin granuloma	0.043	1 Chinese[3]<br>1 USA pt hetero[20]	2
67.	#	R716Q	CGG-CAG	+	LP	NP		1 Turkish[10]	1
68.	#-	R716W	CGG-TGG	+	P	Omenn syndrome		1 pt hetero Italian[23, 32]	1
69.	#-	E722K	CTC GAG-CTC AAG	+	P	SCID, B cell -ve	0.032	2 unrelated hetero pts[19]<br>1 German pt[19, 33]<br>1 pt[7]<br>1 from Russia and 1 from Montenegr	5
70.		E722Q	CTC GAG-CTC CAG			Omenn syndrome with T- B- NK+		1 Russian, Moscow [5]hetero	1
71.	#-	R737H	CGT-CAT	+	P/LP<br>(R737H, R507W pathogenic)	Omenn syndrome	0.043	1 Chinese (who has R108*)[4]<br>1 Turkish hetero[10]<br>1 German hetero who has R507W[2, 15, 16]<br>1 American hetero who has R561C [18, 19]<br>1 Austrian hetero[34]<br>1 from Czech Rep	6
72.	-	R737C	CGT-TGT	+				1 Turkish [22]	1
73.	#-	R759C	CGT-TGT	+	Un.S	Omenn syndrome<br>Combined cellular and humoral immune defects with granuloma	0.043	1 Italian pt[7]	1
74.		R759H	CGT-CAT		Un.S	Combined cellular and humoral immune defects with granuloma			
75.	-	R764H	CGT-CAT	+		RAG1 deficiency			

76.	-	R764P	CGT-CCT			SCID, T & B cell -ve	0.043	1 (hetero cpd)[2]	1
77.	-	R764C	CGT-TGT	+		SCID.		1 Turkish[22]	1
78.	#-	R776W	CGG-TGG	+	LP	SCID, T & B cell -ve<br>Combined cellular and humoral immune defects with granuloma		1Dine Indian American native [35]	1
79.	-	R776Q	CGG-CAG	+		SCID.		1 Turkish[36]	1
80.	#-	R778W	CGG-TGG	+	P	SCID, T & B cell -ve<br>Combined cellular and humoral immune defects with granuloma	0.043	1 Chinese hetero[3]<br>1 (who has R410W[2])<br>1 Italian homo[23]	2
81.	#-	R778Q	CGG-CAG	+	P	SCID<br>Combined cellular and humoral immune defects with granuloma	0.043	1 German hetero[2, 15]	1
82.	-	R778G	CGG-GGG			Omenn syndrome		1 pt hetero in Italy [23]	
83.	-	R841Q	CGG-CAG	+		Autoimmunity, early-onset		1 USA who has F974L[2, 37]	1
84.	#-	R841W	CGG-TGG	+	P	Atypical SCID/Omenn syndrome<br>Alpha/beta T-cell lymphopenia with gamma/delta T-cell expansion, severe cytomegalovirus infection, and autoimmunity		1 homo with autoimmunity[2],<br>1unrelated homo pts [2, 38]<br>1 hetero who has R410Q[2, 19]<br>1 Egyptian[8]	4
85.	#	R841G	CGG-GGG		P	Alpha/beta T-cell lymphopenia with gamma/delta T-cell expansion, severe cytomegalovirus infection, and autoimmunity			
86.	#	p. Ser875=	TCC GAG-TCG GAG		LB	Combined immunodeficiency with skin granulomas, Severe combined immunodeficiency, autosomal recessive, T cell-negative, B cell-negative, NK cell-positive			

87.	#	E876K	TCC GAG-TCC AAG	+	Un.S	Combined cellular and humoral immune defects with granuloma			
88.	#	p. His879=	CAC GAG-CAT GAG	+	LB	Severe combined cellular and humoral immune defects with granulomas, HMR, Alpha/beta T-cell lymphopenia with gamma/delta T-cell expansion, severe cytomegalovirus infection, and autoimmunity,			
89.	#	E880K	CAC GAG-CAC AAG	+	B	NP			
90.	#	R897Q	CGA-CAA	+	LP	NP			
91.	#-	R897*	CGA-TGA	+	P	SCID,<br>B cell -ve		1 Japanese (hetero)[6]<br>1 hetero who has R624[19]	2
92.	#	R918C	CGT-TGT		Un.S	Combined cellular and humoral immune defects with granuloma			
93.	#	R918H	CGT-CAT	+	Un.S	Combined cellular and humoral immune defects with granuloma			
94.	#	T925M	ACG-ATG	+	Un.S	Combined cellular and humoral immune defects with granuloma			
95.	-	R973H	CGC-CAC	+		Omenn syndrome		1 Egyptian[8]<br>1 French [21]<br>1 Asian[29]	3
96.	#-	R973C	CGC-TGC	+	C.P<br>(1LP, 1Un.S)	Immunodeficiency, combined, with granuloma and/or autoimmunity<br>Combined cellular and humoral immune defects with granuloma		1 hetero with autoimmunity[2]	1
97.	#	R973P	CGC-CCC		Un.S	Combined cellular and humoral immune defects with granulom			
98.	#	R975P	CGG-CAG	+	Un.S	Combined cellular and humoral immune defects with granuloma			
99.	#-	R975Q	CGG-CAG	+	P	Omenn syndrome		1 Egyptian homo[8, 39]<br>1 USA hetero who has R396C)[2, 20]	3

								1 who has R396L [2, 18, 19]	
100.	#-	R975W	CGG-TGG	+	Un.S	SCID		1 German (who has R778Q)[2, 15, 16]<br>1 Chinese hetero[40]	2
101.									110
102.	#-	M1V	ATG-GTG		C.P<br>(1 LP, 4 Un.S)	SCID, T & B cell -ve		1 Austria (who has R737H)[34]	1
103.	#	F5L	TTC-TTG		Un.S	Combined immunodeficiency with skin granulomas; Severe combined immunodeficiency, autosomal recessive, T cell-negative, B cell-negative, NK cell-positive			
104.	#	G10V	GGA-GTA		Un.S	Combined immunodeficiency with skin granulomas; Severe combined immunodeficiency, autosomal recessive, T cell-negative, B cell-negative, NK cell-positive			
105.	#	S13A	TCT-GCT		C.P<br>(2 Un.S, 1LB)	Combined cellular and humoral immune defects with granuloma			
106.	#	A14T	TCT GCC- TCT ACC		Un.S	Combined immunodeficiency with skin granulomas<br>Severe combined immunodeficiency, autosomal recessive, T cell-negative, B cell-negative, NK cell-positive			
107.	#	D16G	GAT-GGT		LB	Combined immunodeficiency with skin granulomas, Severe combined immunodeficiency, autosomal recessive, T cell-negative, B cell-negative, NK cell-positive			
108.	#	p. His20=	CAC CCA-CAT CCA		LB	NP			

109.	#	p. Pro21=	CCA-CCT		LB	NP			
110.	#	p. Ser26=	TCA-TCG		LB	NP			
111.	#	W28R	TGG-AGG		Un.S	Combined cellular and humoral immune defects with granuloma			
112.	#	L32V	CTG-GTG		Un.S	Combined cellular and humoral immune defects with granuloma			
113.	#	F33Y	TTC-TAC		Un.S	Combined immunodeficiency with skin granulomas, Severe combined immunodeficiency, autosomal recessive, T cell-negative, B cell-negative, NK cell-positive			
114.	#	V35E	GTG-GAG		Un.S	Combined immunodeficiency with skin granulomas, Severe combined immunodeficiency, autosomal recessive, T cell-negative, B cell-negative, NK cell-positive			
115.	#	Glu39=	GAA-GAG		LB	Combined immunodeficiency with skin granulomas, Severe combined immunodeficiency, autosomal recessive, T cell-negative, B cell-negative, NK cell-positive			
116.	#	P42S	CCT-TCT		Un.S	Combined immunodeficiency with skin granulomas, Severe combined immunodeficiency, autosomal recessive, T cell-negative, B cell-negative, NK cell-positive			
117.	#	E44K	GAA-AAA		Un.S	Combined immunodeficiency with skin granulomas, Severe combined immunodeficiency, autosomal recessive, T cell-			

						negative, B cell-negative, NK cell-positive			
118.	#	p. Lys50=	AAG-AAA		LB	NP			
119.	#	D51V	GAT-GTT		Un.S	HMR, Severe Combined Immune Deficiency			
120.	#	Ser52=	TCC TTT-TCT TTT		LB	Combined immunodeficiency with skin granulomas, Severe combined immunodeficiency, autosomal recessive, T cell-negative, B cell-negative, NK cell-positive			
121.	#	P63S	CCA-TCA		Un.S	Combined immunodeficiency with skin granulomas, Severe combined immunodeficiency, autosomal recessive, T cell-negative, B cell-negative, NK cell-positive			
122.	#	P63A	CCA-GCA		Un.S	Severe combined immunodeficiency, autosomal recessive, T cell-negative, B cell-negative, NK cell-positive, Histiocytic medullary reticulosis			
123.	#	p. Pro 63=	CCA-CCG		B/LB	Omenn syndrome, SCID, HMR, Severe Combined Immune Deficiency			
124.	#	A64V	GCA-GTA		Un.S	Combined cellular and humoral immune defects with granuloma			
125.	#	V65I	GCA GTC- GCA ATC		Un.S	Combined immunodeficiency with skin granulomas, Severe combined immunodeficiency, autosomal recessive, T cell-negative, B cell-negative, NK cell-positive			
126.	#	V65F	GCA GTC- GCA TTC		Un.S	Combined immunodeficiency with skin granulomas, Severe combined immunodeficiency,			

						autosomal recessive, T cell-negative, B cell-negative, NK cell-positive			
127.	#	V65A	GCA GTC-GCA GCC		Un.S	Combined immunodeficiency with skin granulomas, Severe combined immunodeficiency, autosomal recessive, T cell-negative, B cell-negative, NK cell-positive			
128.	#	A69G	GCT-GGT		Un.S	Histiocytic medullary reticulosis, Severe combined immunodeficiency, autosomal recessive, T cell-negative, B cell-negative, NK cell-positive, Combined immunodeficiency due to partial RAG1 deficiency, Combined immunodeficiency with skin granulomas			
129.	#	Q72R	CAG-CGG		Un.S	Combined immunodeficiency with skin granulomas, Severe combined immunodeficiency, autosomal recessive, T cell-negative, B cell-negative, NK cell-positive			
130.	#	Q78E	CAG-GAG		Un.S	Combined cellular and humoral immune defects with granuloma			
131.	#	A83V	GCC-GTC		Un.S	Combined cellular and humoral immune defects with granuloma			
132.	#	H84R	CAC-CGC		B	NP			
133.	#	P85A	CCT-GCT		Un.S	Combined immunodeficiency with skin granulomas, Severe combined immunodeficiency, autosomal recessive, T cell-			

						negative, B cell-negative, NK cell-positive			
134.	#	P85S	CCT-TCT		Un.S	Combined immunodeficiency with skin granulomas, Severe combined immunodeficiency, autosomal recessive, T cell-negative, B cell-negative, NK cell-positive			
135.	#	p. Lys86=	AAG-		LB	Combined immunodeficiency with skin granulomas, Severe combined immunodeficiency, autosomal recessive, T cell-negative, B cell-negative, NK cell-positive			
136.	#	p.Asn94=	AAC-AAT		LB	Combined immunodeficiency with skin granulomas, Severe combined immunodeficiency, autosomal recessive, T cell-negative, B cell-negative, NK cell-positive			
137.	#	G99S	AGA GGC-AGA AGC		LB	NP	0.014	1 homo cpd[2]	1
138.		G99D	GGC-GAC		Un.S	Combined immunodeficiency with skin granulomas, Severe combined immunodeficiency, autosomal recessive, T cell-negative, B cell-negative, NK cell-positive			
139.	-	H103Y	AGA CAT-AGA TAT			SCID		1 homo[41]	1
140.	#	H103R	CAT-CGT		Un.S	Combined immunodeficiency with skin granulomas, Severe combined immunodeficiency, autosomal recessive, T cell-negative, B cell-negative, NK cell-positive			

141.	#	Q104E	CAA-GAA		Un.S	Combined immunodeficiency with skin granulomas, Severe combined immunodeficiency, autosomal recessive, T cell-negative, B cell-negative, NK cell-positive			
142.	#	A105V	GCC-GTC		Un.S	NP			
143.	#	N106K	AAC-AAA		Un.S	Combined immunodeficiency with skin granulomas, Severe combined immunodeficiency, autosomal recessive, T cell-negative, B cell-negative, NK cell-positive			
144.	#	L107F	CTT-TTT		Un.S	Combined cellular and humoral immune defects with granuloma			
145.	#	I113V	ATC-GTC		Un.S	Combined immunodeficiency with skin granulomas, Severe combined immunodeficiency, autosomal recessive, T cell-negative, B cell-negative, NK cell-positive			
146.	#	F118L	TTT-TTA		Un.S	SCID, autosomal recessive, T-cell negative, B-cell negative and NK cell positive			
147.	#	D121A	GAT-GCT		Un.S	Combined cellular and humoral immune defects with granuloma			
148.		E122V	GAG-GTG		Un.S	HMR			
149.	#	R125G	AGG-GGG		Un.S	SCID, autosomal recessive, T-cell negative, B-cell negative and NK cell positive			
150.	#	Arg126=	AGA-AGG		Un.S	SCID, autosomal recessive, T-cell negative, B-cell negative and NK cell positive			

151.	#	P128S	CCA-TCA		Un.S	SCID, autosomal recessive, T-cell negative, B-cell negative and NK cell positive, HMR			
152.	#	V129G	GTC-GGC		Un.S	SCID, autosomal recessive, T-cell negative, B-cell negative and NK cell positive			
153.	#	P132S	CCT-TCT		Un.S	Combined cellular and humoral immune defects with granuloma			
154.	#	Val133=	GTG-GTA		Uns.S	Severe combined immunodeficiency, autosomal recessive, T cell-negative, B cell-negative, NK cell-positive, Combined immunodeficiency with skin granulomas			
155.	-	K136Q	AAA-CAA			Omenn syndrome		1 pt Italy [23]	1
156.	#	G139V	GGC-GTC		Un.S	NP			
157.	#	K144N	AAG-AAT		Un.S	Combined cellular and humoral immune defects with granuloma			
158.	#	p. Lys144=	AAG-AAA		LB	NP			
159.	#	R147T	AGA-ACA		Un.S	Combined immunodeficiency with skin granulomas, SCID, autosomal recessive, T cell-negative, B cell-negative, NK cell-positive			
160.	#	D153E	GAC CTC-GAG CTC		Un.S	Combined immunodeficiency with skin granulomas, SCID, autosomal recessive, T cell-negative, B cell-negative, NK cell-positive			
161.	#	I155V	ATT-GTT		Un.S	Combined immunodeficiency with skin granulomas, SCID, autosomal recessive, T cell-negative, B cell-negative, NK cell-positive			

162.	#	Ala156=	GCC AAG-GCT AAG		LB	Combined immunodeficiency with skin granulomas, Severe combined immunodeficiency, autosomal recessive, T cell-negative, B cell-negative, NK cell-positive			
163.	#	A156V	GCC-GTC		B	RECOMBINATION ACTIVATING GENE 1 POLYMORPHISM			
164.	#	K157R	AAG-AGG		Un.S	Combined immunodeficiency with skin granulomas, Severe combined immunodeficiency, autosomal recessive, T cell-negative, B cell-negative, NK cell-positive			
165.	#	K157N	AAG-AAT		Un.S	Combined immunodeficiency with skin granulomas, Severe combined immunodeficiency, autosomal recessive, T cell-negative, B cell-negative, NK cell-positive			
166.	#	V158I	AAG GTT- AAG ATT		Un.S	Combined immunodeficiency with skin granulomas, Severe combined immunodeficiency, autosomal recessive, T cell-negative, B cell-negative, NK cell-positive			
167.	#	Asp162=	GAT-GAC		LB	Combined immunodeficiency with skin granulomas, Severe combined immunodeficiency, autosomal recessive, T cell-negative, B cell-negative, NK cell-positive			

168.	#	D162E	GAT-GAA		C.P (2Un.S, 1L.B)	Combined immunodeficiency with skin granulomas, Severe combined immunodeficiency, autosomal recessive			
169.	#	p.Ala165=	GCA-GCC		LB	Severe Combined immunodeficiency with skin granulomas, Severe combined immunodeficiency, autosomal recessive, T cell-negative, B cell-negative, NK cell-positive			
170.	#	T173S	ACT-TCT		Un.S	Combined cellular and humoral immune defects with granuloma			
171.	#-	C176F	TGC-TTC		C.P (1LP, 1Un.S)	Combined cellular and humoral immune defects with granuloma			
172.	#	M183I	ATG-ATA		Un.S	Combined cellular and humoral immune defects with granuloma Alpha/beta T-cell lymphopenia with gamma/delta T-cell expansion, severe cytomegalovirus infection, and autoimmunity, HMR,			
173.	#	A190D	GCC-GAC		Un.S	Combined immunodeficiency with skin granulomas; Severe combined immunodeficiency, autosomal recessive, T cell-negative, B cell-negative, NK cell-positive			
174.	#-	C192Y	TGT-TAT		L.B	Omenn syndrom Combined immunodeficiency with skin granulomas, Severe combined immunodeficiency, autosomal recessive, T cell-negative, B cell-negative, NK cell-positive		1 pt who has K136Q in Italy[23]	1
175.	#	E193K	TGT GAG-TGT AAG		B	not provided, HMR, not specified,			

						Severe Combined Immune Deficiency			
176.	#	p. Ph196=	TTC CCG-TTT CCG		LB	Combined cellular and humoral immune defects with granulomas, HMR, Alpha/beta T-cell lymphopenia with gamma/delta T-cell expansion, severe cytomegalovirus infection, and autoimmunity,			
177.	#	p. Arg198=	AGG-CGG		C.P (2Un.S,1LB)	HMR, Severe Combined Immune Deficiency Omenn Syndrome			
178.	-	W204R	TGG-CGG			SCID		1 Iranian homo[11]	1
179.	#	p.His205=	CAC-CAT		LB	Severe combined cellular and humoral immune defects with granulomas, HMR, Alpha/beta T-cell lymphopenia with gamma/delta T-cell expansion, severe cytomegalovirus infection, and autoimmunity,			
180.	#	P209T	CCA-ACA		Un.S	SCID, autosomal recessive, T-cell negative, B-cell negative, NK-cell positive, combined immunodeficiency with skin granuloma			
181.	#	p. Cys211=	TGT-TGC		LB	Combined cellular and humoral immune defects with granulomas, HMR, Alpha/beta T-cell lymphopenia with gamma/delta T-cell expansion, severe cytomegalovirus infection, and autoimmunity,			

182.	#	p. Thr 216=	ACT-ACA		Un.S	Combined immunodeficiency with skin granulomas, Severe combined immunodeficiency, autosomal recessive,			
183.	#	Q227L	CAG-CTG		Un.S	SCID, autosomal recessive, T-cell negative, B-cell negative, NK-cell positive, combined immunodeficiency with skin granuloma			
184.	-	Q231*	CAG-TAG			SCID			
185.	#	R238A	ACT-GCT		Un.S	SCID, autosomal recessive, T-cell negative, B-cell negative, NK-cell positive, combined immunodeficiency with skin granuloma			
186.	#	Q242R	CAA-CGA		C.P (1Un.S, 3B)	SCID, autosomal recessive, T-cell negative, B-cell negative, NK-cell positive, combined immunodeficiency with skin granuloma			
187.	#	R244G	AGA-GGA		NP	NP			
188.	#	A246V	GCC-GTC		LB	NP			
189.	-	Q248*	CAG-TAG			SCID, T & B cell -ve	0.012	1 homo pt with OS[2, 17] 1 homo with T-B- SCID with suspected MFT[23]	1
190.	#	p. Lys250=	AAG-AAA		LB	Combined cellular and humoral immune defects with granulomas, HMR, Alpha/beta T-cell lymphopenia with gamma/delta T-cell expansion, severe cytomegalovirus infection, and autoimmunity,			

191.	#	A267G	GCC-GGC		Un.S	SCID, autosomal recessive, T-cell negative, B-cell negative, NK-cell positive, combined immunodeficiency with skin granuloma				
192.	#	N268S	AAC-AGC		Un.S	Combined immunodeficiency with skin granulomas, Severe combined immunodeficiency, autosomal recessive,				
193.	#	p. His273=	CAT-CAC		LB	Combined cellular and humoral immune defects with granulomas, HMR, Alpha/beta T-cell lymphopenia with gamma/delta T-cell expansion, severe cytomegalovirus infection, and autoimmunity,				
194.	#	S275N	AGT-AAT		Un.S	Combined cellular and humoral immune defects with granuloma				
195.	#	T276A	ACC-GCC		Un.S	Combined immunodeficiency with skin granulomas, Severe combined immunodeficiency, autosomal recessive				
196.	#	K277R	AAG-AGG		Un.S	Combined cellular and humoral immune defects with granuloma			[42]1 hetero in USA (4 pts in the same family)	1
197.	#	p. Lys277=	AAG-AAA		Un.S	Combined immunodeficiency with skin granulomas, Severe combined immunodeficiency, autosomal recessive				
198.	#	P284S	CCA-TCA		Un.S	Combined immunodeficiency with skin granulomas, Severe combined immunodeficiency, autosomal recessive,				
199.	#	P284L	CCA-CTA		Un.S	Combined cellular and humoral immune defects with granuloma				
200.	-	S292P	TCC-CCC			SCID/Wiskott-Aldrich Syndrome				

201.	#	S292F	TCC-TTC		Un.S	Combined immunodeficiency with skin granulomas, Severe combined immunodeficiency, autosomal recessive,			
202.	#	S292C	TCC-TGC		Un.S	SCID, autosomal recessive, T-cell negative, B-cell negative, NK-cell positive, combined immunodeficiency with skin granuloma			
203.	#	C296S	TGT-TCT		Un.S	SCID, autosomal recessive, T-cell negative, B-cell negative, NK-cell positive, combined immunodeficiency with skin granuloma			
204.	#	D302E	GAC CCT-GAA CCT		B	NP			
205.	#	T306I	ACC-ATC		Un.S	SCID, autosomal recessive, T-cell negative, B-cell negative, NK-cell positive, combined immunodeficiency with skin granuloma			
206.	-	C313R	TGC-CGC			SCID, atypical			
207.	-	C313Y	TGC-TAC			SCID			
208.	#	p. Leu318=	CTC-CTT		LB	Severe combined cellular and humoral immune defects with granulomas, HMR, Alpha/beta T-cell lymphopenia with gamma/delta T-cell expansion, severe cytomegalovirus infection, and autoimmunity,			
209.	-	M324V	ATG-GTG			SCID		1 Iranian homo[11]	1
210.	#-	C328Y	TGT-TAT		P	Combined cellular and humoral immune defects with granuloma	0.012	1 homo Italian[18 ,2]	1

						Omenn syndrome			
211.	#	p. Cys331=	TGC CGA-TGT CGA		LB	NP			
212.	#	p. Arg332=	CGA-CGT		LB	Combined cellular and humoral immune defects with granulomas, HMR, Alpha/beta T-cell lymphopenia with gamma/delta T-cell expansion, severe cytomegalovirus infection, and autoimmunity,			
213.	#-	Y333*	TAT-TAA		P	NP			
214.	#	Y333H	TAT-CAT		Un.S	Combined cellular and humoral immune defects with granuloma			
215.	#	C335R	TGC-CGC		LP	Combined immunodeficiency with skin granulomas		[8]1 Egyptian (Novel)	1
216.	#	P337R	CCT-CGT		Un.S	combined immunodeficiency with skin granulomas, Severe combined immunodeficiency, autosomal recessive, T cell-negative, B cell-negative, NK cell-positive			
217.	#	T338P	ACT-CCT		Un.S	Combined cellular and humoral immune defects with granuloma			
218.	#	p. Leu340=	CTG-CTA		Un.S	Combined immunodeficiency with skin granulomas, Severe combined immunodeficiency, autosomal recessive,			
219.	#	p. Val344=	GTG-GTA		LB	Severe combined cellular and humoral immune defects with granulomas, HMR, Alpha/beta T-cell lymphopenia with gamma/delta T-cell expansion, severe cytomegalovirus infection, and autoimmunity,			

220.	#	L354M	CTG-ATG		Un.S	Combined cellular and humoral immune defects with granuloma			
221.	#	M355T	ATG-ACG		C.P (3Un.S, 1LB)	Combined cellular and humoral immune defects with granuloma			
222.	-	C358Y	TGT-TAT			Immunodeficiency, common variable		1 Iran homo[43]	1
223.	#	Pro359=	CCA-CCG		Un.S	Combined immunodeficiency with skin granulomas, Severe combined immunodeficiency, autosomal recessive,			
224.	-	C363Y	TGC-TAC			SCID, atypical			
225.	#	Y372H	TAT-CAT		Un.S	Combined cellular and humoral immune defects with granuloma			
226.	#	H375D	CAC-GAC		Un.S	CID (combined immunodeficiency)		1 German who has [25]R474C	1
227.	#	K383E	AAA-GAA		Un.S	Combined immunodeficiency with skin granulomas, Severe combined immunodeficiency, autosomal recessive,			
228.	#	I385T	ATT-ACT		LB	Combined cellular and humoral immune defects with granuloma			
229.	#	V387A	GTG-GCG		Un.S	Combined immunodeficiency with skin granulomas, Severe combined immunodeficiency, autosomal recessive,			
230.	-	K391E	AAA-GAA			Omenn syndrome	0.03	1 homo compound who [2]has G99S	1
231.	-	G392E	GGG-GAG			Omenn syndrome			
232.	#	G393V	GGC-GTC		C.P (1LB, 2Un.S)	Combined cellular and humoral immune defects with granuloma			
233.	#-	S401P	TCG-CCG		NP	NP			
234.	#-	T403P	ACT-CCT		Un.S	SCID, T & B cell -ve	0.02	[2]1 homo pt	1
235.	#	T403S	ACT-TCT		Un.S				

236.	#	R405G	AGA-GGA		No interpretation	No interpretation			
237.	-	R405I	AGA-ATA			SCID			
238.	#	Q407E	CAG-GAG		L.P	SCID, autosomal recessive, T-cell-negative, B cell-negative, NK cell-positive			
239.		Q407H	CAG -CAC			SCID		1 Egyptian[8]	1
240.	#-	L411P	CTG-CCG		LP	Combined cellular and humoral immune defects with granulomas<br>SCID, T & B cell -ve	0.025	1 homo [2]<br>1 Indian hetero[12]	2
241.	#	p. Arg412=	AGG-AGA		LB	Combined cellular and humoral immune defects with granulomas, HMR, Alpha/beta T-cell lymphopenia with gamma/delta T-cell expansion, severe cytomegalovirus infection, and autoimmunity,			
242.	#	p.Glu413=	GAG-GAA		LB	Severe combined cellular and humoral immune defects with granulomas, HMR, Alpha/beta T-cell lymphopenia with gamma/delta T-cell expansion, severe cytomegalovirus infection, and autoimmuni			
243.	#	p.Leu416=	CTG-TTG		LB	Severe combined cellular and humoral immune defects with granulomas, HMR, Alpha/beta T-cell lymphopenia with gamma/delta T-cell expansion, severe cytomegalovirus infection, and autoimmuni			

244.	#	K419R	AAA-AGA		Un.S	Combined cellular and humoral immune defects with granuloma			
245.	#	p.Ala420=	GCC TTT-GCT TTT		LB	Combined cellular and humoral immune defects with granulomas, HMR, Alpha/beta T-cell lymphopenia with gamma/delta T-cell expansion, severe cytomegalovirus infection, and autoimmuni			
246.	#	p.Ala422=	GCT-GCA		LB	Combined cellular and humoral immune defects with granulomas, HMR, Alpha/beta T-cell lymphopenia with gamma/delta T-cell expansion, severe cytomegalovirus infection, and autoimmuni			
247.	#	G428R	GGA-AGA		Un.S	SCID, autosomal recessive, T-cell-negative, B cell-negative, NK cell-positive			
248.	#-	D429G	GAT-GGT		P	Omenn syndrome HMR	0.022	1 Yugoslavian [19 ,18 ,2] hetero pt with fs	1
249.	#-	M435V	ATG-GTG		P/LP	Omenn syndrome Alpha/beta T-cell lymphopenia with gamma/delta T-cell expansion, severe cytomegalovirus infection, and autoimmunity, combined cellular and humoral immune defects with granulomas, HMR, Combined cellular and humoral immune defects with granulomas	0.019	3 unrelated hetero 1 who has R699W, 2 with R559S [2], one of the later in both [2, 19] 1 homo cpd from Tenn, USA [2, 44] 1 hetero Newcastle,UK[45]	4

250.	#	A441S	CTG GCT- CTG TCT		Un.S	SCID, autosomal recessive, T-cell-negative, B cell-negative, NK cell-positive			
251.	-	R443K	AGG-AAG			SCID/ Wiskott-Aldrich Syndrome		1 Asian[29]	1
252.	#-	R449K	AGG-AAG		C.P (1Un.S, 5B, 1LB)	Omenn Sybdrome SCID, autosomal recessive, T-cell-negative, B cell-negative, NK cell-positive	0.043	1 pt who has R404Q in the same copy and R764P in . [2]the other 1 pt homo in Italy[23]	2
253.	-	R449S	AGG-AGT			Omenn syndrome		1 pt Uk Newcastle [45]	1
254.	#	Q450*	CAA-TAA		L.P	Combined immunodeficiency with skin granuloma			
255.	#	E453D	GAG-GAT		Un.S	SCID, autosomal recessive, T-cell-negative, B cell-negative, NK cell-positive			
256.	#-	L454Q	CTG-CAG		Un.S	Omnn syndrome Combined cellular and humoral immune defects with granuloma,	0.025	1 Israel homo[46 ,2]	1
257.	-	E455K	CTG GAG-CTG AAG			RAG1 deficiency			
258.	#	A456D	GCC-GAC		C.P (2Un.S, 1LB)	SCID, autosomal recessive, T-cell-negative, B cell-negative, NK cell-positive,HMR			
259.	-	A472V	GCC-GTC			SCID	0.027		
260.	#-	S480G	AGC-GGC		LP	SCID, autosomal recessive, T-cell-negative, B cell-negative, NK cell-positive			
261.	#	p.Ser480=	AGC TGC-AGT TGC		LB	Combined immunodeficiency with skin granulomas, Severe combined immunodeficiency, autosomal recessive,			
262.	#	p.Tyr484=	TAC CAC-TAT CAC		LB	Combined immunodeficiency with skin granulomas, Severe			

						combined immunodeficiency, autosomal recessive,			
263.	#	M487R	ATG-AGG		P	SCID, autosomal recessive, T-cell-negative, B cell-negative, NK cell-positive			
264.	#	R489S	AGG-AGC		Un.S	Combined cellular and humoral immune defects with granuloma,			
265.	#	T490A	ACT-GCT		Un.S	Combined cellular and humoral immune defects with granuloma,			
266.	#	V491A	GTG-GCG		Un.S	SCID, autosomal recessive, T-cell-negative, B cell-negative, NK cell-positive			
267.	#	I494V	ATC-GTC		Un.S	Combined immunodeficiency due to partial RAG1 deficiency.			
268.	#	Q498H	CAG-CAC		Un.S	SCID, autosomal recessive, T-cell-negative, B cell-negative, NK cell-positive			
269.	-	L506F	CTT-TTT			CD4+T lymphoaenia	0.025	1 Netherland patient who has R474C[2, 26]	1
270.	#	p. Asn508=	AAT-AAC		LB	NP			
271.	-	E510*	GCT GAG-GCT TAG			SCID		[3]2 unrelated Chinese pts (hetero one with fs and the other who has E669K)	2
272.	-	L514R	CTG-CGG			Atypical SCID			
273.	#	p.Leu514=	CTG-CTA		LB	Combined cellular and humoral immune defects with granulomas, HMR, Alpha/beta T-cell lymphopenia with gamma/delta T-cell expansion, severe cytomegalovirus infection, and autoimmunity,			

274.	#	P515L	CCA-CTA		Un.S	Combined cellular and humoral immune defects with granulomas			
275.	-	G516A	GGC-GCC			Immunodeficiency, combined, with granuloma and/or autoimmunity	0.014	[2]1 pt hetero	1
276.	#	H518Q	CAC-CAA		Un.S	SCID, autosomal recessive, T-cell-negative, B cell-negative, NK cell-positive			
277.	#	H519Q	CAC-CAG		Un.S	SCID, autosomal recessive, T-cell-negative, B cell-negative, NK cell-positive			
278.	#	F520S	TTT-TCT		Un.S	Combined cellular and humoral immune defects with granuloma,			
279.	#	F520L	TTT-CTT		Un.S	Combined cellular and humoral immune defects with granuloma,			
280.	#-	W522C	TGG-TGT		P	Omenn syndrome/Atypical SCID Combined cellular and humoral immune defects with granuloma,	0.005	1 hetero who has E722K[19]<br>1 hetero who has R973C[2]<br>1 hetero with fs [2, 47]	3
281.	#	P525S	CCT-TCT		B	NP			
282.		L526R	CTG-CGG					1 pt[48]	1
283.	#	p. Leu526=	CTG-CTA		B	NP			
284.	-	D539V	GAT-GTT			Omenn syndrome	0.022		
285.	#	G540R	GAT GGG-GAT AGG		Un.S	NP			
286.	#	L544I	CTA-ATA		Un.S	Combined cellular and humoral immune defects with granulomas			

287.	#	p. Leu544=	CTG-CTA		LB	NP			
288.	#	S546F	TCC-TTC		Un.S	Combined cellular and humoral immune defects with granuloma,			
289.	#	p.Ser546=	TCC-TCT		LB	Severe combined cellular and humoral immune defects with granulomas, HMR, Alpha/beta T-cell lymphopenia with gamma/delta T-cell expansion, severe cytomegalovirus infection, and autoimmunity,			
290.	-	D550N	GAT-AAT			SCID			
291.	#	D554G	GAC-GGC		Un.S	Combined cellular and humoral immune defects with granuloma,			
292.	#	D554V	GAC-GTC		Un.S	Combined cellular and humoral immune defects with granulomas			
293.	#	p. Thr555=	ACC-ACT		LB	NP			
294.	#	K558N	AAG-AAC		Un.S	SCID, autosomal recessive, T-cell negative, B-cell negative, NK-cell positive, combined immunodeficiency with skin granuloma, HMR			
295.	-	R559S	AGG-AGC<br>AGG-AGT		P	Omenn syndrome<br>SCID, autosomal recessive, T-cell negative, B-cell negative, NK-cell positive, combined immunodeficiency with skin granuloma		1 Japanese who has R897*[6]<br>2 unrelated pts who has M435[2, 19]<br>3 hetero with fs (Russia, Vladivostok)[5]<br>1 Egyptian homo[8]	5

296.		A565P	GCT-CCT			SCID		[8]1 Egyptian (Novel)	1
297.	#	D576V	GAC-GTC		Un.S	SCID, autosomal recessive, T-cell negative, B-cell negative, NK-cell positive, combined immunodeficiency with skin granuloma			
298.	#	M581T	ATG-ACG		Un.S	SCID, autosomal recessive, T-cell negative, B-cell negative, NK-cell positive, combined immunodeficiency with skin granuloma			
299.	#	D587E	GAT-GAG		Un.S	SCID, autosomal recessive, T-cell negative, B-cell negative, NK-cell positive, combined immunodeficiency with skin granuloma			
300.	#-	Y589*	TAC CTG-TAG CTG		P	SCID, autosomal recessive, T-cell negative, B-cell negative, NK-cell positive, combined immunodeficiency with skin granuloma		1 Turkish[22]<br>1Italian [19, 23]	1
301.	#	p.Leu590=	CTG-CTT		Un.S	SCID			
302.	#	V596M	ACT GTG-ACT ATG		Un.S	SCID, autosomal recessive, T-cell negative, B-cell negative, NK-cell positive, combined immunodeficiency with skin granuloma, HMR			
303.	-	S601P	TCT-CCT			Immunodeficiency, combined, with granuloma and/or autoimmunity	0.041	[2]1 pt who has G516A	1
304.	#	S601A	TCT-GCT		Un.S	SCID, autosomal recessive, T-cell negative, B-cell negative, NK-cell positive, combined immunodeficiency with skin granuloma			

305.	-	C602W	TGT-TGG			SCID, T & B cell -ve		1 Italian pt homo[23]	1
306.	#	p.Asp603=	GAT-GAC		LB	Severe combined cellular and humoral immune defects with granulomas, HMR, Alpha/beta T-cell lymphopenia with gamma/delta T-cell expansion, severe cytomegalovirus infection, and autoimmunity,			
307.	#	M605I	ATG-ATC		No Interpretation for single variant	No Interpretation			
308.	#	p.Asp607=	GAC-GAT		LB	Combined cellular and humoral immune defects with granulomas, HMR, Alpha/beta T-cell lymphopenia with gamma/delta T-cell expansion, severe cytomegalovirus infection, and autoimmunity,			
309.	#	V608M	GGA GTG-GGA ATG		Un.S	SCID, autosomal recessive, T-cell negative, B-cell negative, NK-cell positive, combined immunodeficiency with skin granuloma			
310.	#	S609G	AGT-GGT		Un.S	SCID, autosomal recessive, T-cell negative, B-cell negative, NK-cell positive, combined immunodeficiency with skin granuloma			
311.	#-	H612R	CAT-CGT		Un.S	SCID, autosomal recessive, T-cell negative, B-cell negative, NK-cell positive, combined immunodeficiency with skin granuloma	0.026	1 homo[49 ,2]<br>1 German homo[16, 50]	2
312.		K621E	AAG-GAG			SCID		[8]1 pt	1

313.	#-	A622P	AAG GCA-AAG CCA		Un.S	SCID, T & B cell -ve		1 Italian pt who has R474S[23]	1
314.	#	A622T	GCA-ACA		Un.S	SCID, autosomal recessive, T-cell negative, B-cell negative, NK-cell positive, combined immunodeficiency with skin granuloma			
315.	#	F625L	TTT-TTG		Un.S	SCID, autosomal recessive, T-cell negative, B-cell negative, NK-cell positive, combined immunodeficiency with skin granuloma			
316.	-	S626*	TCA-TAA			T- B-	0.041	1 homo pt[2] Maternal engraftment	1
317.	-	M630T	ATG-ACG			Omenn syndrome		1 Chinese hetero who R404W[3]has	1
318.	#	T633A	ACT-GCT		Un.S	SCID, autosomal recessive, T-cell negative, B-cell negative, NK-cell positive, combined immunodeficiency with skin granuloma			
319.	#	A635S	ATT GCC-ATT TCC		Un.S	SCID, autosomal recessive, T-cell negative, B-cell negative, NK-cell positive, combined immunodeficiency with skin granuloma			
320.	#	H636R	CAC-CGC		Un.S	Combined cellular and humoral immune defects with granuloma			
321.	#	K642Q	AAA-CAA		Un.S	Combined cellular and humoral immune defects with granuloma			
322.	-	N650K	AAC TCT-AAG TCT			SCID, T & B cell -ve		1 pt hetero Italian[23]	1
323.	#	p.Leu658=	TTG-TTA		LB	Combined cellular and humoral immune defects with granulomas, HMR, Alpha/beta T-cell lymphopenia			l

						with gamma/delta T-cell expansion, severe cytomegalovirus infection, and autoimmunity,			
324.	#	A663E	GCA-GAA		Un.S	NP			
325.	#	E665K	GAT GAG-GAT AAG		Un.S	NP			
326.	-	S666P	TCT-CCT			SCID		1 Chinese who has [3] W896R	1
327.	-	E669G	GAG-GGG			Omenn syndrome		1 pt hetero [19]	1
328.	#	p.Leu671=	CTC-CTT		LB	Combined cellular and humoral immune defects with granulomas, HMR, Alpha/beta T-cell lymphopenia with gamma/delta T-cell expansion, severe cytomegalovirus infection, and autoimmunity,			
329.	#	P677A	CCT-GCT		Un.S	Combined cellular and humoral immune defects with granulomas			
330.	#	I679V	ATT-GTT		Un.S	Combined cellular and humoral immune defects with granuloma,			
331.	-	E681*	GCT GAG- GCT TAG		Un.S	SCID			
332.	#	K686E	AAG-GAG		Un.S	SCID, autosomal recessive, T-cell negative, B-cell negative, NK-cell positive, combined immunodeficiency with skin granuloma			
333.	#	S687N	AGC-AAC		Un.S	Combined cellular and humoral immune defects with granuloma,			

334.	#	E689K	AGT GAA-AGT AAA		Un.S	SCID, autosomal recessive, T-cell negative, B-cell negative, NK-cell positive, combined immunodeficiency with skin granuloma			
335.	-	L692P	CTT-CCT			SCID			
336.	#	p.Leu694=	CTG-CTA		LB	Combined cellular and humoral immune defects with granuloma Severe immunodeficiency, autosomal recessive, T-cell negative, B-cell negative, NK cell-positive			
337.	#	G696D	GGC-GAC		Un.S	SCID, autosomal recessive, T-cell negative, B-cell negative, NK-cell positive, combined immunodeficiency with skin granuloma			
338.	#	F701Y	TTC-TAC		Un.S	SCID, autosomal recessive, T-cell negative, B-cell negative, NK-cell positive, combined immunodeficiency with skin granuloma			
339.	#	I704V	ATC-GTC		Un.S	HMR, Severe Combined Immune Deficiency			
340.	#	p.Arg706=	AGG-AGA		LB	Combined cellular and humoral immune defects with granulomas, HMR, Alpha/beta T-cell lymphopenia with gamma/delta T-cell expansion, severe cytomegalovirus infection, and autoimmunity,			
341.	#	G707R	AGG GGC-AGG CGC		No interpretation for the single variant	NP			
342.	-	G707D	GGC-GAC			SCID			

343.	#	p.Thr708=	ACC GGC-ACT GGC		LB	Combined cellular and humoral immune defects with granulomas, HMR, Alpha/beta T-cell lymphopenia with gamma/delta T-cell expansion, severe cytomegalovirus infection, and autoimmunity,			
344.	#	G709A	GGC-GCC		Un.S	SCID, autosomal recessive, T-cell negative, B-cell negative, NK-cell positive, combined immunodeficiency with skin granuloma			
345.	-	G709D	GGC-GAC			Omenn syndrome		1 pt homo in Italy[23]<br>1 pt from Slovenia	2
346.	#	L714F	CTT-TTT		Un.S	SCID, autosomal recessive, T-cell negative, B-cell negative, NK-cell positive, combined immunodeficiency with skin granuloma			
347.	#	L721V	CTC-GTC		Un.S	NP			
348.	#	p.Leu721=	CTC-GTC		LB	Combined cellular and humoral immune defects with granulomas, HMR, Alpha/beta T-cell lymphopenia with gamma/delta T-cell expansion, severe cytomegalovirus infection, and autoimmunity,			
349.	#	p.Val727=	GTC		LB	Combined cellular and humoral immune defects with granulomas, HMR, Alpha/beta T-cell lymphopenia with gamma/delta T-cell expansion, severe			

						cytomegalovirus infection, and autoimmunity,			
350.	#-	Y728H	TAC-CAC		No interpretation	Common variable immunodeficiency			
351.	#	I729V	ATT-GTT		Un.S	SCID, autosomal recessive, T-cell negative, B-cell negative, NK-cell positive, combined immunodeficiency with skin granuloma			
352.	#	I729L	ATT-CTT		Un.S	SCID, autosomal recessive, T-cell negative, B-cell negative, NK-cell positive, combined immunodeficiency with skin granuloma			
353.	#	I729M	ATT-ATG		Un.S	SCID, autosomal recessive, T-cell negative, B-cell negative, NK-cell positive, combined immunodeficiency with skin granuloma			
354.	-	C730F	TGT-TTT			Omenn syndrome	0.012	1 who has R699Q[2] 1 French who has R474H[21]	2
355.	-	C730Y	TGT-TAT			Omenn syndrome		[3]1 Chinese homo	1
356.		C730*	TGT-TGA					,3]1 Chinese hetero with fs [31	1
357.	-	T731I	ACT-ATT			SCID		1 Iranian homo [51 ,11]	1
358.	-	L732P	CTT-CCT			SCID		1 pt who has R474C[2, 28]	1
359.	-	L732F	CTT-TTT			Omenn syndrome		[21]1 French	1
360.	#	C733*	TGT-TGA		LP	Not provided			
361.	#	p. Asp734=	GAT-GAC		LB	combined immunodeficiency with skin granulomas, Severe			

						combined immunodeficiency, autosomal recessive, T cell-negative, B cell-negative, NK cell-positive			
362.	#	A735V	GCC-GTC		Un.S	Combined cellular and humoral immune defects with granuloma,			
363.	#	A740G	GCC-GGC		Un.S	SCID, autosomal recessive, T-cell negative, B-cell negative, NK-cell positive, combined immunodeficiency with skin granuloma			
364.	#	p. Val745=	GTC-GTT		LB	Combined cellular and humoral immune defects with ,granulomas, HMR Alpha/beta T-cell lymphopenia with gamma/delta T-cell expansion, severe cytomegalovirus infection, and ,autoimmunity			
365.	#	F746C	TTC-TGC		Un.S	SCID, autosomal recessive, T-cell negative, B-cell negative, NK-cell positive, combined immunodeficiency with skin granuloma			
366.	#	H747P	CAC-CCC		LP	NP			
367.	#	I749V	ATA-GTA		Un.S	SCID, autosomal recessive, T-cell negative, B-cell negative, NK-cell positive, combined immunodeficiency with skin granuloma			
368.	#	T750S	ACC-TCC		Un.S	SCID, autosomal recessive, T-cell negative, B-cell negative, NK-cell positive, combined			

						immunodeficiency with skin granuloma			
369.	#-	H753L	CAT-CTT		Un.S	Omenn syndrome		1 hetero who has [19]R474H	1
370.	#-	H753R	CAT-CGT		Un.S	Omenn syndrome/Atypical SCID			
371.	-	Y768*	TAC CAT-TAA CAT			SCID, T & B cell -ve			
372.	-	E770K	CAT GAG-CAT AAG			SCID		1 pt homo[28 ,2] (Maternal engraftment)	1
373.	#-	E774*	GAA GAA-GAA TAA		P	SCID, B cell -ve		[33 ,19]1 German who has E722K	1
374.		L775Q	CTG-CAG					R973H[29]1 pt who has	1
375.	#	p.Leu775=	CTG-CTA		LB	Combined cellular and humoral immune defects with granulomas, HMR, Alpha/beta T-cell lymphopenia with gamma/delta T-cell expansion, severe cytomegalovirus infection, and autoimmunity,			
376.	-	V779M	CGG GTG-CGG ATG			Omenn syndrome			
377.	#	S783*	TCA-TGA		P/LP	Combined cellular and humoral immune defects with granulomas, HMR, Alpha/beta T-cell lymphopenia with gamma/delta T-cell expansion, severe cytomegalovirus infection, and autoimmunity,			
378.	-	P786L	CCT-CTT			Omenn syndrome		1 who has R559S autoimmunity[2] With	1
379.	#	p.Pro792=	CCT-CCA		LB	Combined immunodeficiency with skin granulomas, Severe			

						combined immunodeficiency, autosomal recessive, T cell-negative, B cell-negative, NK cell-positive			
380.	-	I794T	ATA-ACA			SCID		1 Indian descent hom[52]	1
381.	#	H798R	CAC-CGC		LP	Combined cellular and humoral immune defects with granulomas			
382.	#	C799Y	TGT-TAT		Un.S	Combined cellular and humoral immune defects with granulomas			
383.	#	G802S	ATT GGC-ATTAGC		Un.S	Combined immunodeficiency with skin granulomas, Severe combined immunodeficiency, autosomal recessive, T cell-negative, B cell-negative, NK cell-positive			
384.	#	A804V	GCA-GTA		L.P	SCID, autosomal recessive, T cell-negative, B cell-negative, NK cell-positive, Combined immunodeficiency with skin granulomas			
385.	#	K809R	AAG-AGG		Un.S	HMR, Combined cellular and humoral immune defects with granuloma, SCID			
386.	#	I810V	ATC-GTG		Un.S	Combined cellular and humoral immune defects with granulomas			
387.	-	Q812*	CAG-TAG			Omenn syndrome		1 Egyptian homo[8, 39]	1
388.	#	E814D	GAG-GAT		C.P (1LP, 1Un.S)	not provided, Combined cellular and humoral immune defects with granulomas			
389.	#	I815T	ATA-ACA		Un.S	Combined cellular and humoral immune defects with granuloma			

390.	#	I815M	ATA-ATG		Un.S	Combined cellular and humoral immune defects with granulomas			
391.	#	G816W	ATA GGG- ATA TGG		Un.S	Combined cellular and humoral immune defects with granulomas			
392.	#	p.Gly816=	GGG-GGA		LB	Combined immunodeficiency with skin granulomas, Severe combined immunodeficiency, autosomal recessive, T cell-negative, B cell-negative, NK cell-positive			
393.	#	p. Glu817=	GAA-GAG		B/LB	NP			
394.	#	K820E	AAG-GAG		Un.S	Combined cellular and humoral immune defects with granulomas			
395.	#-	K820R	AAG-AGG		B	Non-Hodgkin lymphoma, Increased risk, association with	0.03	1 Iranian homo [11, 12]<br>1 USA [53]	2
396.	#	N823S	AAT-AGT		Un.S	Combined cellular and humoral immune defects with granuloma			
397.	#	N823T	AAT-ACT		Un.S	SCID			
398.	#	S825A	TCC-GCC		Un.S	Combined cellular and humoral immune defects with granuloma			
399.	#	p. Glu827=	GAG-GAA		LB	Combined immunodeficiency with skin granulomas, Severe combined immunodeficiency, autosomal recessive, T cell-negative, B cell-negative, NK cell-positive			
400.		R829S	AGG-AGC			Classical OS		1 Ukraine, Kalush[5, 23]	1
401.	#	K830*	AAA-TAA		LP	RAG1-Related Disorders		1 who has R829S[5, 23]	1

402.	#	K830T	AAA-ACA		Un.S	Combined cellular and humoral immune defects with granulomas			
403.	#	A834S	CAG GCC-CAG TCC		Un.S	Combined cellular and humoral immune defects with granuloma			
404.	-	L836V	CTG-GTG			SCID			
405.	#	p. Leu836=	CTG-TTG		LB	Severe Combined immunodeficiency with skin granulomas, Severe combined immunodeficiency, autosomal recessive, T cell-negative, B cell-negative, NK cell-positive			
406.	#	H839Y	CAT-TAT		Un.S	Combined cellular and humoral immune defects with granuloma			
407.	#	H839L	CAT-CTT		Un.S	Combined cellular and humoral immune defects with granuloma			
408.	#	K843E	AAG-GAG		Un.S	Combined cellular and humoral immune defects with granuloma			
409.	-	K847E	AAA-GAA			SCID		1 chinese pt who has R108*[3]	1
410.	#	M852L	ATG-TTG		Un.S	Combined cellular and humoral immune defects with granulomas			
411.		G854D	GGC-GAC					1 pt who has R561[29]	1
412.	#-	N855I	AAC-ATC		NP	SCID		1 homo pt with MFT[19]	1
413.	#	N855S	AAC-AGC		LP	NP			
414.	-	A857V	GCC-GTC			Omenn syndrome		1 Chinese homo[3]	1
415.	#	p. Ala857=	GCC AGG-GCT AGG		C.P (2Un.S, 1B, 2LB)	NP			

416.	#	D867A	GAT-GCT		Un.S	Combined cellular and humoral immune defects with granulomas			
417.	#	A868V	GCA-GTA		C.P (1LP, 1Un.S)	SCID, Combined cellular and humoral immune defects with granuloma			
418.	#	V869F	GCA GTT-GCA TTT		Un.S	Combined cellular and humoral immune defects with granuloma			
419.	#	Val869=	GTT-GTC		LB	Severe Combined immunodeficiency with skin granulomas, Severe combined immunodeficiency, autosomal recessive, T cell-negative, B cell-negative, NK cell-positive			
420.	-	L872*	TTA-TAA			SCID, T & B cell -ve		1 pt homo[19]<br>1 pt hetero with fs[19]	2
421.	-	L885R	CTG-CGG			Omenn syndrome		1 hetero (two Japanese siblings who have R396C)[6]	1
422.		L885I	CTG-ATG			SCID with MFT		1 pt homo [19]	1
423.	#	D887N	ATG GAT-ATG AAT		B/LB	HMR & Severe combined immunodeficiency			
424.	#	Y889H	TAC-CAC		Un.S				
425.	-	W896R	TGG-CGG			SCID	0.005	1 Chinese pt homo[2, 54]<br>1 Chinese pt who has S666P[3, 31]	2
426.	-	W896*	TGG-TGA			T- B- SCID		1 Iranian homo[11, 51]	1
427.	-	C900*	TGC-TGA			Omenn syndrome		1 pt who has E669G[19]	1
428.		K903*	AAA-TAA					1 pt from Slovenia with Omenn Syndrome	1
429.	#	p. Cys905=	TGC CCA-TGT CCA		LB	Combined cellular and humoral immune defects with granulomas			

430.	#	Q911H	CAG-CAC		Un.S	Combined cellular and humoral immune defects with granulomas			
431.	#-	Y912C	TAC-TGC		P	Omenn syndrome HMR	0.008	1 pt who has R396C[2, 18, 19]	1
432.	#	Q917*	CAG-TAG		LP	Combined immunodeficiency due to partial **RAG1** deficiency			
433.	#	p. Gln917=	CAG-CAA		C.P	SCID/Omenn Syndrome HMR			
434.	#	K926T	AAG-ACG		Un.S	Combined immunodeficiency with skin granulomas, Severe combined immunodeficiency, autosomal recessive, T cell-negative, B cell-negative, NK cell-positive			
435.	#	p. Gly933=	GGA-GGG		C.P (1LB, 2Un.S)	NP			
436.	#-	Y938*	TAT-TAG		P	SCID, B cell -ve #-		1 German pt hetero with fs[19]	1
437.	#	T942S	ACC-TCC		Un.S	Severe combined immunodeficiency, autosomal recessive, T cell-negative, B cell-negative, NK cell-positive, Combined immunodeficiency with skin granulomas			
438.	#	T942I	ACC-ATC		Un.S	HMR & Severe combined immunodeficiency Omenn Syndrome			
439.	#	p. Ala944=	GCC CAT-GCG CAT		LB	Severe combined cellular and humoral immune defects with granulomas, HMR, Alpha/beta T-cell lymphopenia with gamma/delta T-cell expansion, severe cytomegalovirus infection, and autoimmunity,			

440.	#	H945R	CAT-CGT		Un.S	Combined immunodeficiency with skin granulomas, Severe combined immunodeficiency, autosomal recessive, T cell-negative, B cell-negative, NK cell-positive			
441.	#	E948D	GAA-GAT		Un.S	Combined immunodeficiency with skin granulomas, Severe combined immunodeficiency, autosomal recessive, T cell-negative, B cell-negative, NK cell-positive			
442.	#	G954V	GGC-GTC		Un.S	Combined cellular and humoral immune defects with granuloma Severe immunodeficiency, autosomal recessive, T-cell negative, B-cell negative, NK cell-positive			
443.	-	I956T	ATT-ACT			Omenn syndrome		1 Italian pt homo[2, 23] 1 from Poland with fs	1
444.	-	G957V	GGG-GTG			SCID			
445.	#	Ala958=	GCA-GCG		LB	Combined cellular and humoral immune defects with granulomas, HMR, Alpha/beta T-cell lymphopenia with gamma/delta T-cell expansion, severe cytomegalovirus infection, and autoimmunity,			
446.	-	W959*	TGG-TAG			SCID	0.005	2 pts homo with MFT[2, 19]	2
447.	#	A960S	GCA-TCA		Un.S	Combined cellular and humoral immune defects with granulomas			

448.	#	p. Ala960=	GCA-GCG		B/LB	Omenn Syndrome Severe Combined Immune Deficiency		[12]	
449.	-	E965*	AAT GAG-AAT TAG			SCID			
450.	#	S966T	TCT-ACT		Un.S	Combined cellular and humoral immune defects with granulomas			
451.	#	N968K	AAC AAA-AAA AAA		C.P (1LP, 1Un.S)	HMR			
452.	#	p. Phe971=	TTT-TTC		LB	Severe combined cellular and humoral immune defects with granulomas, HMR, Alpha/beta T-cell lymphopenia with gamma/delta T-cell expansion, severe cytomegalovirus infection, and autoimmunity,			
453.	-	F974L	TTC-CTC			Autoimmunity/early onset	0.013	1 pt who has R841Q[2, 37]	1
454.	#-	Q981P	CAG-CCG		P	Alpha/beta T-cell lymphopenia with gamma/delta T-cell expansion, severe cytomegalovirus infection-#, and autoimmunity	0.012	[38 ,2]1 pt homo	1
455.	#	S982Y	TCC-TAC		Un.S	Combined cellular and humoral immune defects with granulomas			
456.	#	p. Tyr985=	TAT-TAC		B/LB	NP			
457.	#	V990I	GAT GTC-GAT ATC		Un.S	HMR & Severe combined immunodeficiency			
458.	#	p.Val 990=	GTC CTG-GTA CTG		LB	Combined cellular and humoral immune defects with granulomas, HMR, Alpha/beta T-cell lymphopenia with gamma/delta T-cell			

						expansion, severe cytomegalovirus infection, and autoimmunity,			
459.	-	K992R	AAA-AGA			Omenn syndrome		1 pt in Italy who has R778G [23]	1
460.	#-	K992E	AAA-GAA		P	Omenn syndrome<br>Combined cellular and humoral immune defects with granulomas<br>Severe combined immunodeficiency, autosomal recessive, T cell-negative, B cell-negative, NK cell-positive	0.03	1 hetero in USA (4 pts in the same family who also have K277R)[42]<br>1 French who has R624H[21]<br>1 homo pt	3
461.	#	H993Q	CAC CAC-CAA CAC		Un.S	Combined cellular and humoral immune defects with granulomas			
462.	#	H994R	CAC-CGC		C.P<br>(1LP, 1Un.S)	Combined cellular and humoral immune defects with granuloma			
463.	#	L996F	TTG-TTT		Un.S	Combined cellular and humoral immune defects with granulomas			
464.	#	p. Tyr997=	TAC ACC-TAT ACC		LB	Combined cellular and humoral immune defects with granulomas, HMR, Alpha/beta T-cell lymphopenia with gamma/delta T-cell expansion, severe cytomegalovirus infection, and autoimmunity,			
465.	-	Y1001*	TAC CTC-TAG CTC			Omenn syndrome		1 Chinese pt who has R699W[3]	1
466.	#	Q1003H	CAG-CAT		Un.S	Severe combined cellular and humoral immune defects with granulomas			

467.	#	p.Lys1004=	AAG-AAA		LB	Severe combined cellular and humoral immune defects with granulomas, HMR, Alpha/beta T-cell lymphopenia with gamma/delta T-cell expansion, severe cytomegalovirus infection, and autoimmunity,			
468.	#-	M1006V	ATG-GTG		C.P (2Un.S, 3LB)	Omenn syndrome with aniridia	0.03	1 Iranian homo[2, 11] 1 pt homo cpd who has M435V[2, 44]	2
469.	#	N1007D	AAT-GAT		Un.S	Severe combined cellular and humoral immune defects with granulomas			
470.	#	H1009Y	CAT- TAT		Un.S	Combined cellular and humoral immune defects with granuloma Severe immunodeficiency, autosomal recessive, T-cell negative, B-cell negative, NK cell-positive			
471.	#	p.Thr1014=	ACC TCT-ACT TCT		LB	Combined cellular and humoral immune defects with granulomas, HMR, Alpha/beta T-cell lymphopenia with gamma/delta T-cell expansion, severe cytomegalovirus infection, and autoimmunity,			
472.	#	T1018I	ACC-ATC		Un.S	Severe combined immunodeficiency, autosomal recessive, T cell-negative, B cell-negative, NK cell-positive, Combined immunodeficiency with skin granulomas			
473.	#	M1019T	ATG-ACG		Un.S	Severe combined immunodeficiency, autosomal recessive, T cell-negative, B cell-			

						negative, NK cell-positive, Combined immunodeficiency with skin granulomas			
474.	#	P1028L	CCA-CTA		Un.S	Severe combined immunodeficiency, autosomal recessive, T cell-negative, B cell-negative, NK cell-positive, Combined immunodeficiency with skin granulomas			
475.	#	P1028Q	CCA-CAA		Un.S	Combined cellular and humoral immune defects with granulomas.			
476.	#	p.Pro1028=	CCA-CCG		LB	Severe combined immunodeficiency, autosomal recessive, T cell-negative, B cell-negative, NK cell-positive|Combined immunodeficiency with skin granulomas			
477.	#	G1030V	GGC-GTC		Un.S	Combined immunodeficiency with skin granulomas, Severe combined immunodeficiency, autosomal recessive, T cell-negative, B cell-negative, NK cell-positive			
478.	#	E1036Q	CTG GAA-CTG CAA		Un.S	Combined immunodeficiency with skin granulomas, Severe combined immunodeficiency, autosomal recessive, T cell-negative, B cell-negative, NK cell-positive			

# refers to mutation found in https://www.ncbi.nlm.nih.gov/clinvar?term=179615[MIM]

- refers to mutation found in http://www.hgmd.cf.ac.uk/ac/gene.php?gene=RAG1
#- refers to mutation found in both sites.
+ means mutation is mediated by methylation
¥MRF is the raw mutation rate frequency, high MRF score proves a higher possibility of occurrence. MRFmax=0.043 and MRFmin=0.004. Values of MRF are taken from [1].
Tweenty mutations with MRFmax, nineteen of them are of CpG mutations and eighteen of them are mediated by methylatio

References

1. Lawless, D., et al., *Predicting the Occurrence of Variants in RAG1 and RAG2.* J Clin Immunol, 2019. **39**(7): p. 688-701.
2. Lee, Y.N., et al., *A systematic analysis of recombination activity and genotype-phenotype correlation in human recombination-activating gene 1 deficiency.* J Allergy Clin Immunol, 2014. **133**(4): p. 1099-108.
3. Bai, X., et al., *Clinical, immunologic, and genetic characteristics of RAG mutations in 15 Chinese patients with SCID and Omenn syndrome.* Immunol Res, 2016. **64**(2): p. 497-507.
4. Chi, Z.H., et al., *Targeted high-throughput sequencing technique for the molecular diagnosis of primary immunodeficiency disorders.* Medicine (Baltimore), 2018. **97**(40): p. e12695.
5. Sharapova, S.O., et al., *Molecular Characteristics, Clinical and Immunologic Manifestations of 11 Children with Omenn Syndrome in East Slavs (Russia, Belarus, Ukraine).* J Clin Immunol, 2016. **36**(1): p. 46-55.
6. Kato, M., et al., *Omenn syndrome--review of several phenotypes of Omenn syndrome and RAG1/RAG2 mutations in Japan.* Allergol Int, 2006. **55**(2): p. 115-9.
7. Cassani, B., et al., *Defect of regulatory T cells in patients with Omenn syndrome.* J Allergy Clin Immunol, 2010. **125**(1): p. 209-16.
8. Meshaal, S.S., et al., *Phenotypical heterogeneity in RAG-deficient patients from a highly consanguineous population.* Clin Exp Immunol, 2019. **195**(2): p. 202-212.
9. Sharapova, S.O., et al., *The Clinical and Genetic Spectrum of 82 Patients With RAG Deficiency Including a c. 256_257delAA Founder Variant in Slavic Countries.* Frontiers in Immunology, 2020. **11**: p. 900.
10. Erman, B., et al., *Investigation of Genetic Defects in Severe Combined Immunodeficiency Patients from Turkey by Targeted Sequencing.* Scand J Immunol, 2017. **85**(3): p. 227-234.
11. Safaei, S., et al., *IL7R and RAG1/2 genes mutations/polymorphisms in patients with SCID.* Iran J Allergy Asthma Immunol, 2011. **10**(2): p. 129-32.

12. Crestani, E., et al., *RAG1 reversion mosaicism in a patient with Omenn syndrome.* J Clin Immunol, 2014. **34**(5): p. 551-4.
13. Noordzij, J.G., et al., *The immunophenotypic and immunogenotypic B-cell differentiation arrest in bone marrow of RAG-deficient SCID patients corresponds to residual recombination activities of mutated RAG proteins.* Blood, 2002. **100**(6): p. 2145-52.
14. Tabori, U., et al., *Detection of RAG mutations and prenatal diagnosis in families presenting with either T-B- severe combined immunodeficiency or Omenn's syndrome.* Clin Genet, 2004. **65**(4): p. 322-6.
15. Schuetz, C., et al., *An immunodeficiency disease with RAG mutations and granulomas.* N Engl J Med, 2008. **358**(19): p. 2030-8.
16. Schuetz, C., et al., *SCID patients with ARTEMIS vs RAG deficiencies following HCT: increased risk of late toxicity in ARTEMIS-deficient SCID.* Blood, 2014. **123**(2): p. 281-9.
17. Kutukculer, N., et al., *Novel mutations and diverse clinical phenotypes in recombinase-activating gene 1 deficiency.* Ital J Pediatr, 2012. **38**: p. 8.
18. Villa, A., et al., *Partial V(D)J recombination activity leads to Omenn syndrome.* Cell, 1998. **93**(5): p. 885-96.
19. Villa, A., et al., *V(D)J recombination defects in lymphocytes due to RAG mutations: severe immunodeficiency with a spectrum of clinical presentations.* Blood, 2001. **97**(1): p. 81-8.
20. Avila, E.M., et al., *Highly variable clinical phenotypes of hypomorphic RAG1 mutations.* Pediatrics, 2010. **126**(5): p. e1248-52.
21. Corneo, B., et al., *Identical mutations in RAG1 or RAG2 genes leading to defective V(D)J recombinase activity can cause either T-B-severe combined immune deficiency or Omenn syndrome.* Blood, 2001. **97**(9): p. 2772-6.
22. Patiroglu, T., H.H. Akar, and M. Van Der Burg, *Three faces of recombination activating gene 1 (RAG1) mutations.* Acta Microbiol Immunol Hung, 2015. **62**(4): p. 393-401.
23. Sobacchi, C., et al., *RAG-dependent primary immunodeficiencies.* Hum Mutat, 2006. **27**(12): p. 1174-84.
24. Gruber, T.A., et al., *Clinical and genetic heterogeneity in Omenn syndrome and severe combined immune deficiency.* Pediatr Transplant, 2009. **13**(2): p. 244-50.
25. Schroder, C., et al., *Evaluation of RAG1 mutations in an adult with combined immunodeficiency and progressive multifocal leukoencephalopathy.* Clin Immunol, 2017. **179**: p. 1-7.
26. Kuijpers, T.W., et al., *Idiopathic CD4+ T lymphopenia without autoimmunity or granulomatous disease in the slipstream of RAG mutations.* Blood, 2011. **117**(22): p. 5892-6.
27. Chen, K., et al., *Autoimmunity due to RAG deficiency and estimated disease incidence in RAG1/2 mutations.* J Allergy Clin Immunol, 2014. **133**(3): p. 880-2 e10.
28. Asai, E., et al., *Analysis of mutations and recombination activity in RAG-deficient patients.* Clin Immunol, 2011. **138**(2): p. 172-7.
29. Luk, A.D.W., et al., *Family History of Early Infant Death Correlates with Earlier Age at Diagnosis But Not Shorter Time to Diagnosis for Severe Combined Immunodeficiency.* Front Immunol, 2017. **8**: p. 808.
30. Ehl, S., et al., *A variant of SCID with specific immune responses and predominance of gamma delta T cells.* J Clin Invest, 2005. **115**(11): p. 3140-8.

31. Zhang, Z.Y., et al., *Clinical characteristics and molecular analysis of three Chinese children with Omenn syndrome.* Pediatr Allergy Immunol, 2011. **22**(5): p. 482-7.
32. Cavadini, P., et al., *AIRE deficiency in thymus of 2 patients with Omenn syndrome.* J Clin Invest, 2005. **115**(3): p. 728-32.
33. Schwarz, K., et al., *RAG mutations in human B cell-negative SCID.* Science, 1996. **274**(5284): p. 97-9.
34. Geier, C.B., et al., *Leaky RAG Deficiency in Adult Patients with Impaired Antibody Production against Bacterial Polysaccharide Antigens.* PLoS One, 2015. **10**(7): p. e0133220.
35. Xiao, Z., et al., *A novel missense RAG-1 mutation results in T-B-NK+ SCID in Athabascan-speaking Dine Indians from the Canadian Northwest Territories.* Eur J Hum Genet, 2009. **17**(2): p. 205-12.
36. Karaca, N.E., et al., *Diverse phenotypic and genotypic presentation of RAG1 mutations in two cases with SCID.* Clin Exp Med, 2009. **9**(4): p. 339-42.
37. Henderson, L.A., et al., *Expanding the spectrum of recombination-activating gene 1 deficiency: a family with early-onset autoimmunity.* J Allergy Clin Immunol, 2013. **132**(4): p. 969-71 e1-2.
38. de Villartay, J.P., et al., *A novel immunodeficiency associated with hypomorphic RAG1 mutations and CMV infection.* J Clin Invest, 2005. **115**(11): p. 3291-9.
39. Meshaal, S., et al., *Mutations in Recombination Activating Gene 1 and 2 in patients with severe combined immunodeficiency disorders in Egypt.* Clin Immunol, 2015. **158**(2): p. 167-73.
40. Shen, J., et al., *A Novel RAG1 Mutation in a Compound Heterozygous Status in a Child With Omenn Syndrome.* Front Genet, 2019. **10**: p. 913.
41. Khan, T.A., et al., *Novel RAG1 mutation and the occurrence of mycobacterial and Chromobacterium violaceum infections in a case of leaky SCID.* Microb Pathog, 2017. **109**: p. 114-119.
42. Strauss, K.A., et al., *Clinical application of DNA microarrays: molecular diagnosis and HLA matching of an Amish child with severe combined immune deficiency.* Clin Immunol, 2008. **128**(1): p. 31-8.
43. Abolhassani, H., et al., *A hypomorphic recombination-activating gene 1 (RAG1) mutation resulting in a phenotype resembling common variable immunodeficiency.* J Allergy Clin Immunol, 2014. **134**(6): p. 1375-1380.
44. Sheehan, W.J., et al., *Novel presentation of Omenn syndrome in association with aniridia.* J Allergy Clin Immunol, 2009. **123**(4): p. 966-9.
45. Gennery, A.R., et al., *Omenn's syndrome occurring in patients without mutations in recombination activating genes.* Clin Immunol, 2005. **116**(3): p. 246-56.
46. Dalal, I., et al., *Novel mutations in RAG1/2 and ADA genes in Israeli patients presenting with T-B-SCID or Omenn syndrome.* Clin Immunol, 2011. **140**(3): p. 284-90.
47. De Ravin, S.S., et al., *Hypomorphic Rag mutations can cause destructive midline granulomatous disease.* Blood, 2010. **116**(8): p. 1263-71.
48. Matangkasombut, P., et al., *Lack of iNKT cells in patients with combined immune deficiency due to hypomorphic RAG mutations.* Blood, 2008. **111**(1): p. 271-4.

49. Felgentreff, K., et al., *Clinical and immunological manifestations of patients with atypical severe combined immunodeficiency.* Clin Immunol, 2011. **141**(1): p. 73-82.
50. John, T., et al., *Unrelated Hematopoietic Cell Transplantation in a Patient with Combined Immunodeficiency with Granulomatous Disease and Autoimmunity Secondary to RAG Deficiency.* J Clin Immunol, 2016. **36**(7): p. 725-32.
51. Fazlollahi, M.R., et al., *Clinical, Laboratory, and Molecular Findings for 63 Patients With Severe Combined Immunodeficiency: A Decade s Experience.* J Investig Allergol Clin Immunol, 2017. **27**(5): p. 299-304.
52. Dhingra, N., et al., *Severe combined immunodeficiency caused by a new homozygous RAG1 mutation with progressive encephalopathy.* Hematol Oncol Stem Cell Ther, 2014. **7**(1): p. 44-9.
53. Hill, D.A., et al., *Risk of non-Hodgkin lymphoma (NHL) in relation to germline variation in DNA repair and related genes.* Blood, 2006. **108**(9): p. 3161-7.
54. Zhang, J., et al., *Novel RAG1 mutation in a case of severe combined immunodeficiency.* Pediatrics, 2005. **116**(3): p. e445-9.

Table S2: List of CpG mutations in RAG1 coding sequence

	Mutation	Codon change	Clinical Significance	Conditions in ncbi	In HMGD	Citation	no
1. #-	R73H	CGC-CAC	CP (1LP, 2Un.S)	HMR, Primary immunodeficiency, HMR, Recombinase activating gene 2 deficiency, Atypical SCID due to complete RAG1/2 deficiency	OS	1 Japanese [1, 2],<br>1 South Asia[3]	2
2. #-	R73C	CGC-TGC	P/LP	SCID disease, SCID, autosomal recessive, T cell-, B cell-, NK cell-+, Combined cellular and humoral immune defects with granulomas	SCID	1 Chinese pt[4]	1
3. #	R123H	CGC-CAC	Un.S	SCID, autosomal recessive, T cell-, B cell-, NK cell-+, HMR, Combined cellular and humoral immune defects with granulomas, SCID, autosomal recessive, T cell-, B cell-, NK cell-+, HMR, Combined cellular and humoral immune defects with granulomas, SCID, autosomal recessive, T cell-, B cell-, NK cell-+			
4. #	R123C	CGC-TGC	C.P (6Un.S,1LB)	SCID, autosomal recessive, T cell-, B cell-, NK cell-+, Combined cellular and humoral immune defects with granulomas, SCID, autosomal recessive, T cell-, B cell-, NK cell-+, HMR			
5. #-	R148*	CGA-TGA	P	SCID, autosomal recessive, T cell-, B cell-, NK cell-+, Combined cellular and humoral immune defects with granulomas	OS	1 homo Egy(2siblings)[5, 6]<br>1 South Asian [3]	2
6. #	R148Q	CGA-CAA	Un.S	SCID, autosomal recessive, T cell-, B cell-, NK cell-+, Combined cellular and humoral immune defects with granulomas			
7. #-	R159C	CGC-TGC	P	SCID, autosomal recessive, T cell-, B cell-, NK cell-+, Combined cellular and humoral immune defects with granulomas		1 Asian[3]	1
8. #	R159H	CGC-CTC	LP	SCID, autosomal recessive, T cell-, B cell-, NK cell-+, Combined cellular and humoral immune defects with granulomas			
9. #-	R229W	CGG-TGG	C.P (1P, 1Un.S)	Primary immunodeficiency, HMR, Recombinase activating gene 2 deficiency, SCID, autosomal recessive, T cell-, B cell-, NK cell-+, Combined cellular and humoral immune defects	Atypical SCID\OS	2 homo (3 pts; 2 of them are siblings) [1, 7]<br>5 homo (1 of them arab) and 1 hetero Israel pts [1, 8]<br>1 Italian[1, 9]	12

				with granulomas, SCID, autosomal recessive, T cell-, B cell-, NK cell-+		1 Asian[3]<br>2 homo Iran[1, 10]		
10.	#-	R229Q	CGG-CAG	C.P (1P, 1LP, 1Un.S)	SCID, B cell-, Combined cellular and humoral immune defects with granulomas, SCID, autosomal recessive, T cell-, B cell-, NK cell-+, HMR, Recombinase activating gene 2 deficiency, SCID, autosomal recessive, T cell-, B cell-, NK cell-+, Primary immunodeficiency, HMR	SCID, B^-	1 homo pt[1, 7, 11]<br>1 hetero[1, 7, 12]<br>4 Egyptian unrelated pts[6], 1 Egyptian[5, 6]<br>1 French hetero who has R39G[1, 13]<br>1 Iranian[14]	9	
11.	-#	R229P	CGG-CCG	LP	Combined cellular and humoral immune defects with granulomas	SCID	1 homo USA [15]	1	
12.	#	R229L	CGG-CTG	LP	SCID, B cell-, Combined cellular and humoral immune defects with granulomas, SCID, autosomal recessive, T cell-, B cell-, NK cell-				
13.	#	R300C	CGT-CAT	Un.S	SCID, autosomal recessive, T cell-, B cell-, NK cell-+, Combined cellular and humoral immune defects with granulomas				
14.	#	R300H	CGT-TGT	L.B	SCID, autosomal recessive, T cell-, B cell-, NK cell-+, Combined cellular and humoral immune defects with granulomas				
15.	#	p. Pro441=	CCC GCC-CCT GCC	LB	SCID, autosomal recessive, T cell-, B cell-, NK cell-+, SCID, autosomal recessive, T cell-, B cell-, NK cell-+, Combined cellular and humoral immune defects with granulomas,				
16.	#	R464H	CGC-CAC	C.P (3Un.S,1LB)	SCID, autosomal recessive, T cell-, B cell-, NK cell-+				
17.	#	R464C	CGC-TGC	Un.S	SCID, autosomal recessive, T cell-, B cell-, NK cell-+				
18.	#	R506H	CGT-CAT	Un.S	SCID, autosomal recessive, T cell-, B cell-, NK cell-+				
19.	#	R506C	CGT-TGT	Un.S	SCID, autosomal recessive, T cell-, B cell-, NK cell-+				
20.								28	
21.	#	M1T	ATG-ACG	P/LP	Primary immunodeficiency, Recombinase activating gene 2 deficiency, not provided				
22.	-	Q4*	CAG-TAG			SCID			
23.	#	M5K	ATG-AAG	Un.S	Severe Combined Immune Deficiency, HMR, Combined cellular and				

					humoral immune defects with granulomas, SCID, autosomal recessive, T cell-, B cell-, NK cell-+			
24.	#	V8I	ACA GTC-ACA ATC	B/LB	Combined cellular and humoral immune defects with granulomas, SCID, autosomal recessive, T cell-, B cell-, NK cell-+, SCID, autosomal recessive, T cell-, B cell-, NK cell-+, HMR			
25.	#	p. Val8=	GTC AGT-GTT AGT	LB	SCID, autosomal recessive, T cell-, B cell-, NK cell-+, Combined cellular and humoral immune defects with granulomas			
26.	#	S9G	AGT-GGT	Un.S	SCID, autosomal recessive, T cell-, B cell-, NK cell-+, Combined cellular and humoral immune defects with granulomas			
27.	#	I12T	ATA-ACA	LB	SCID, autosomal recessive, T cell-, B cell-, NK cell-+, Combined cellular and humoral immune defects with granulomas			
28.	#	p. Ala13=	GCC TTA-GCA TTA<br>GCC TTA-GCT TTA	LB	SCID, autosomal recessive, T cell-, B cell-, NK cell-+, Combined cellular and humoral immune defects with granulomas			
29.	-	Q16*	CAG-TAG			SCID, B^-	1 Netherland[1, 16]	1
30.	#	p. Pro17=	CCA-CCG	LB	SCID, autosomal recessive, T cell-, B cell-, NK cell-+, Combined cellular and humoral immune defects with granulomas			
31.	#	G18V	CCA GGC-CCA TGC	Un.S	SCID, autosomal recessive, T cell-, B cell-, NK cell-+, Combined cellular and humoral immune defects with granulomas			
32.	#	p. Leu21=	CTG-CTA	C.P (2Un.S,1LB)	SCID, autosomal recessive, T cell-, B cell-, NK cell-+, HMR, Combined cellular and humoral immune defects with granulomas, SCID, autosomal recessive, T cell-, B cell-, NK cell-+			
33.	#	N23S	AAT-AGT	Un.S	SCID, autosomal recessive, T cell-, B cell-, NK cell-+, Combined cellular and humoral immune defects with granulomas			
34.	#	D25G	GAT-GGT	Un.S	HMR, SCID, autosomal recessive, T cell-, B cell-, NK cell-+			
35.		F29S	TTC-TCC			SCID	1 homo Egyptian[6]	1
36.	-	G32D	GGA-GAA			RAG deficien		

						cy		
37.	#-	G35A	GGC-GCC	C.P (1P, 1LP, 1Un.S)	SCID disease, Primary immunodeficiency, Recombinase activating gene 2 deficiency, Atypical SCID due to complete RAG1/2 deficiency, SCID, autosomal recessive, T cell-, B cell-, NK cell-+, Combined cellular and humoral immune defects with granulomas	Hyper-IgM syndrome	2 homo and 1 hetero pts[1]<br>1 pt homo (USA)[1, 15]	3
38.	#-	G35V	GGC-GTC	P/LP	Primary immunodeficiency, HMR, Recombinase activating gene 2 deficiency, SCID, autosomal recessive, T cell-, B cell-, NK cell-+	SCID, B^-	5 unrelated homo Egyptian pts[6]<br>1 French homo[1, 13]<br>1 Italian homo[1, 9]<br>1 Israel homo[1, 17]<br>3 homo Arab pts in Israel[1, 8]<br>3 South Asia[3]	14
39.	#	P37L	CCC-CTC	Un.S	HMR			
40.	#	p.Pro37=	CCC AAA-CCA AAA	LB	SCID, autosomal recessive, T cell-, B cell-, NK cell-+, Combined cellular and humoral immune defects with granulomas			
41.	#-	R39G	AGA-GGA	LP	Recombinase activating gene 2 deficiency, SCID, autosomal recessive, T cell-, B cell-, NK cell-+, Primary immunodeficiency, SCID, B cell-, HMR	SCID, B^-	1 French pt [1, 13, 18]	1
42.	#	R39I	AGA-ATA	Un.S	SCID, autosomal recessive, T cell-, B cell-, NK cell-+, Combined cellular and humoral immune defects with granulomas			
43.	#	S40Y	TCC TGC-TCA TGC	Un.S	SCID, autosomal recessive, T cell-, B cell-, NK cell-+, Combined cellular and humoral immune defects with granulomas			
44.	#-	C41W	TGC CCC-TGG CCC	P/LP	Recombinase activating gene 2 deficiency, SCID, autosomal recessive, T cell-, B cell-, NK cell-+, Primary immunodeficiency, HMR	OS	1 Italian hetero cpd[7, 19]	1
45.	#	G44*	ACT GGA- ACT TGG	P	SCID, autosomal recessive, T cell-, B cell-, NK cell-+, Primary immunodeficiency			
46.	#	H47Q	CAT-CAG	Un.S	SCID, autosomal recessive, T cell-, B cell-, NK cell-+, Primary immunodeficiency, HMR			
47.	#	H47L	CAT-CTT	Un.S	SCID, autosomal recessive, T cell-, B cell-, NK cell-+, Primary			

				immunodeficiency, HMR				
48. #	N53H	AAC-CAC	Un.S	SCID, autosomal recessive, T cell-, B cell-, NK cell-+, Primary immunodeficiency				
49. #	p.Lys58=	ACA-ACG	C.P (2Un.S,1LB)	SCID, autosomal recessive, T cell-, B cell-, NK cell-+, HMR				
50. #	Thr60=	AAG-AAA	LB	not provided				
51. -	F62V	TTC-GTC			SCID	1 USA homo pt[1, 15]		
52. #-	F62L	TTC TCT-TTA TCT	LP	Primary immunodeficiency, Combined immunodeficiency with skin granulomas, Recombinase activating gene 2 deficiency	SCID			
53. #-	D65Y	AAG GAT-AAG TAT	P/LP	not provided, Primary immunodeficiency, Recombinase activating gene 2 deficiency, SCID, autosomal recessive, T cell-, B c SCID, autosomal recessive, T cell-, B cell-, NK cell-+, Combined cellular and humoral immune defects with granulomas ell-, NK cell-+	SCID, T$^-$ &B$^-$	1 homo Israel pt [1, 20]	1	
54. #	p.Asp65=	GAT-GAC	LB	SCID, autosomal recessive, T cell-, B cell-, NK cell-+, Combined cellular and humoral immune defects with granulomas				
55. #	p.Leu69=	CTC CCT-CTG CCT	LB	SCID, autosomal recessive, T cell-, B cell-, NK cell-+, Combined cellular and humoral immune defects with granulomas				
56. #	p.Pro71=	CCT-CCC	LB	SCID, autosomal recessive, T cell-, B cell-, NK cell-+, Combined cellular and humoral immune defects with granulomas				
57. #-	T77N	ACT-AAT	LP	Primary immunodeficiency, Combined immunodeficiency with skin granulomas, Recombinase activating gene 2 deficiency, Combined cellular and humoral immune defects with granulomas	SCID	1 German pt[1, 21]	1	
58. #	F80I	TTC-ATC	Un.S	SCID, autosomal recessive, T cell-, B cell-, NK cell-+, Primary immunodeficiency				
59. #	K81R	AAA-AGA	Un.S	SCID, autosomal recessive, T cell-, B cell-, NK cell-+, Combined cellular and humoral immune defects				

					with granulomas				
60.	#	H89Y	CAT-TAT	Un.S	SCID, autosomal recessive, T cell-, B cell-, NK cell-+, Combined cellular and humoral immune defects with granulomas				
61.	#	Q90E	CAT-GAT	Un.S	SCID, autosomal recessive, T cell-, B cell-, NK cell-+, Combined cellular and humoral immune defects with granulomas				
62.	#	Y91*	TAC ATC-TAA ATC	LP	NP				
63.	#	p.Ile92=	ATC ATC-ATA ATC	LB	SCID, autosomal recessive, T cell-, B cell-, NK cell-+, Combined cellular and humoral immune defects with granulomas				
64.	#-	G95R	CAT GGA-CAT AGA	Un.S	SCID, autosomal recessive, T cell-, B cell-, NK cell-+, Combined cellular and humoral immune defects with granulomas	SCID	1 homo Egyptian[1, 6]<br>1 hetero Israel[1, 8]<br>1 hetero Israel[1, 17]<br>1 hetero USA[1, 22]	4	
65.	#	G95R	CAT GGA-CAT CGA	P/LP	Inborn errors of immunity/RAG2 deficiency/HMR				
66.	#	p.Gly 96=	GGG-GGA	LB	Not provided				
67.	#	M110L	ATG-CTG	C.P (1LP,2Un.S)	SCID disease, Primary immunodeficiency, Recombinase activating gene 2 deficiency		1 USA[15]	1	
68.	#	p.Ile112=	ATT-ATC	L.B	SCID, autosomal recessive, T cell-, B cell-, NK cell-+, Combined cellular and humoral immune defects with granulomas				
69.	#	K118R	AAA-AGA	Un.S	Not specified				
70.	#	T121I	ACT-ATT	Un.S	SCID, autosomal recessive, T cell-, B cell-, NK cell-+, Combined cellular and humoral immune defects with granulomas				
71.	#	p.Arg123=	CGC-CGT	LB	SCID, autosomal recessive, T cell-, B cell-, NK cell-+, Combined cellular and humoral immune defects with granulomas				
72.	-	K127*	AAA-TAA			SCID	1 homo Egyptian[1, 6]<br>2 homo Saudi[1, 23]	3	
73.	-	G139S	TAT GGT-TAT AGT				1 Iranian [14]	1	
74.	#	H140R	CAT-CGT	Un.S	SCID, autosomal recessive, T cell-, B cell-, NK cell-+, Combined cellular and humoral immune defects with granulomas				
75.	#	p. Asn143=	AAT-AAC	LB	SCID, autosomal recessive, T cell-, B cell-, NK cell-+, Combined cellular and humoral immune defects				

				with granulomas				
76. #	V144A	GTG-GCG	Un.S	SCID, autosomal recessive, T cell-, B cell-, NK cell-+, Combined cellular and humoral immune defects with granulomas				
77. #	V145A	GTG-GCG	Un.S	SCID, autosomal recessive, T cell-, B cell-, NK cell-+, Combined cellular and humoral immune defects with granulomas				
78. #	p. Val145=	GTG-GTA	LB	SCID, autosomal recessive, T cell-, B cell-, NK cell-+, Combined cellular and humoral immune defects with granulomas				
79. #	G149E	GGG-GAG	Un.S	SCID, autosomal recessive, T cell-, B cell-, NK cell-+, Combined cellular and humoral immune defects with granulomas				
80. #	p. Gly149=	GGG-GGA	LB	SCID, autosomal recessive, T cell-, B cell-, NK cell-+, Combined cellular and humoral immune defects with granulomas				
81. #	Glycine 153=	GGT-GGC	LB	Not provided				
82. #	L155P	CTC-CCC	C.P (1LP, 1Un.S)	Not provided				
83. #-	G157V	GGA-GTA	LP	Primary immunodeficiency, HMR, Recombinase activating gene 2 deficiency	OS	1 homo Israel pt[1, 20]	1	
84. #	G157A	GGA-GCA	LP	SCID, autosomal recessive, T cell-, B cell-, NK cell-+, Combined cellular and humoral immune defects with granulomas				
85. #	G157R	TTT GGA- TTT AGA	LP	SCID, autosomal recessive, T cell-, B cell-, NK cell-+, Combined cellular and humoral immune defects with granulomas				
86. #	Arg159=	CGC TCA-CGT TCA	C.P (2Un.S,2LB)	SCID, autosomal recessive, T cell-, B cell-, NK cell-+, Combined cellular and humoral immune defects with granulomas, SCID, autosomal recessive, T cell-, B cell-, NK cell-+, HMR				
87. #-	S160L	TCA-TTA	C.P (1LP, 1Un.S)	Primary immunodeficiency, HMR, Recombinase activating gene 2 deficiency, not provided	OS	1 Italian pt[1, 9]	1	
88. #	p. Ser160=	TCA-TCG	LB	SCID, autosomal recessive, T cell-, B cell-, NK cell-+, Combined cellular and humoral immune defects				

				with granulomas				
89. #	p. Try161=	TAC-TAT	LB	SCID, autosomal recessive, T cell-, B cell-, NK cell-+, Combined cellular and humoral immune defects with granulomas				
90. #	p.Pro163=	CCT-CCG	LB	SCID, autosomal recessive, T cell-, B cell-, NK cell-+, Combined cellular and humoral immune defects with granulomas				
91. #	H166R	CAC-CGC	Un.S	SCID, autosomal recessive, T cell-, B cell-, NK cell-+, Combined cellular and humoral immune defects with granulomas				
92. #	R167S	AGA-AGC	C.P (1LP, 1Un.S)	SCID, autosomal recessive, T cell-, B cell-, NK cell-+, Combined cellular and humoral immune defects with granulomas				
93. #	N173S	AAT-AGT	LP					
94. -	C178*	TGC-TGA			SCID			
95. #-	P180H	CCC-CAC	P/LP	Recombinase activating gene 2 deficiency, Primary immunodeficiency, Atypical SCID due to complete RAG1/2 deficiency, not provided	RAG2 deficiency			
96. #	C181R	TGT-CGT	Un.S	SCID, autosomal recessive, T cell-, B cell-, NK cell-+, Combined cellular and humoral immune defects with granulomas				
97. #-	F183L	TTC-CTC	L.P	Not provided	SCID			
98.	p. Asp186=	GAT-GAC	LB	SCID, autosomal recessive, T cell-, B cell-, NK cell-+, Combined cellular and humoral immune defects with granulomas				
99. #	E188Q	TTT GAA-TTT CAA	Un.S	SCID, autosomal recessive, T cell-, B cell-, NK cell-+, Combined cellular and humoral immune defects with granulomas				
100. #	p. Glu188=	GAA-GAG	LB	SCID, autosomal recessive, T cell-, B cell-, NK cell-+, Combined cellular and humoral immune defects with granulomas				
101. #	p. Thr193=	ACA-ACG	LB	SCID, autosomal recessive, T cell-, B cell-, NK cell-+, Combined cellular and humoral immune defects with granulomas				
102.	S194*	TCA-TAA		SCID				
103. #-	Y195D	TAC-GAC	LP	Primary immunodeficiency, Recombinase activating gene 2 deficiency				

104. #	Y195H	TAC-CAC	Un.S	SCID, autosomal recessive, T cell-, B cell-, NK cell-+, Combined cellular and humoral immune defects with granulomas				
105. #	p. Try195=	TAC ATT-TAT ATT	LB	SCID, autosomal recessive, T cell-, B cell-, NK cell-+, Combined cellular and humoral immune defects with granulomas				
106. #	E199*	CCA GAA- CCA TAA	P	Combined cellular and humoral immune defects with granulomas, SCID, autosomal recessive, T cell-, B cell-, NK cell-+				
107. #	G203E	GGG-GAG	LP	Common variable immunodeficiency				
108. -	S205Y	TCT-TAT			ID, T^-			
109. -	F206C	TTT-TGT			OS			
110. #	I210T	ATT-ACT	P	Common variable immunodeficiency				
111. #-	T215I	ACC-ATC	C.P (1Un.S, 5B, 2LB)	Recombinase activating gene 2 deficiency, SCID, autosomal recessive, T cell-, B cell-, NK cell-+, Primary immunodeficiency, HMR, SCID, B cell-, Combined cellular and humoral immune defects with granulomas, SCID, autosomal recessive, T cell-, B cell-, NK cell-+, HMR, not specified, SCID, autosomal recessive, T cell-, B cell-, NK cell-+, not provided	SCID	1 Egyptian homo [1, 6]<br>4 unrelated Egyptians homo [5, 6]<br>1 homo Israel[1, 8]	6	
112. #	p.Ile216=	ATC-ATA	LB	SCID, autosomal recessive, T cell-, B cell-, NK cell-+, Combined cellular and humoral immune defects with granulomas				
113. #	p.Tyr217=	TAC-TAT	LB	SCID, autosomal recessive, T cell-, B cell-, NK cell-+, Combined cellular and humoral immune defects with granulomas				
114. #	p.Ala225=	GCC-GCG	LB	SCID, autosomal recessive, T cell-, B cell-, NK cell-+, Combined cellular and humoral immune defects with granulomas				
115. #	N226S	AAT-AGT	Un.S	SCID, autosomal recessive, T cell-, B cell-, NK cell-+, HMR				
116. #	p.Asp227=	AAT-AAC	LB	SCID, autosomal recessive, T cell-, B cell-, NK cell-+, Combined cellular and humoral immune defects with granulomas				
117. #	I228F	ATC-AGC	Un.S	SCID, autosomal recessive, T cell-, B cell-, NK cell-+,				

				Combined cellular and humoral immune defects with granulomas,HMR				
118.	#	R237S	AGG-AGT	Un.S	SCID, autosomal recessive, T cell-, B cell-, NK cell-+, Combined cellular and humoral immune defects with granulomas			
119.	#	L240F	CTT-TTT	Un.S	SCID, autosomal recessive, T cell-, B cell-, NK cell-+			
120.	#	L240P	CTT-CCT	Un.S	SCID, autosomal recessive, T cell-, B cell-, NK cell-+, Combined cellular and humoral immune defects with granulomas			
121.	#	Pro241=	CCC CTG-CCA CTG<br>CCC CTG-CCG CTG	LB	SCID, autosomal recessive, T cell-, B cell-, NK cell-+, Combined cellular and humoral immune defects with granulomas			
122.	#	p.Leu242=	CTG-TTG	LB	SCID, autosomal recessive, T cell-, B cell-, NK cell-+, Combined cellular and humoral immune defects with granulomas			
123.	#	P245S	CCA-TCA	Un.S	SCID, autosomal recessive, T cell-, B cell-, NK cell-+, Combined cellular and humoral immune defects with granulomas			
124.	#	V247E	GTG-GAG	Un.S	SCID, autosomal recessive, T cell-, B cell-, NK cell-+			
125.	#	p.Val247=	GTG-GTA	B	Combined cellular and humoral immune defects with granulomas, SCID, autosomal recessive, T cell-, B cell-, NK cell-+, SCID, autosomal recessive, T cell-, B cell-, NK cell-+, HMR, not specified			
126.	#-	P253R	CCA-CGA	Un.S	Primary immunodeficiency, HMR, Recombinase activating gene 2 deficiency	OS	1 Italian homo [1, 9]	1
127.	#	S259F	TCC-TCT	Un.S	SCID, autosomal recessive, T cell-, B cell-, NK cell-+, Combined cellular and humoral immune defects with granulomas			
128.	#	p. Ser260=	AGT-AGC	LB	SCID, autosomal recessive, T cell-, B cell-, NK cell-+, Combined cellular and humoral immune defects with granulomas			
129.	#	p. Leu262=	ATC-ATT	LB	SCID, autosomal recessive, T cell-, B cell-, NK cell-+, Combined cellular and humoral immune defects with granulomas			
130.	#	p. Leu263=	CTG-CTA<br>CTG-TTG	LB	SCID, autosomal recessive, T cell-, B cell-, NK cell-+, Combined cellular and			

				humoral immune defects with granulomas				
131. #	N268H	AAT-CAT	Un.S	SCID, autosomal recessive, T cell-, B cell-, NK cell-+, Combined cellular and humoral immune defects with granulomas				
132. #	N268S	AAT-AGT	Un.S	SCID, autosomal recessive, T cell-, B cell-, NK cell-+, HMR				
133. #	E270G	GAA-GGA	Un.S	SCID, autosomal recessive, T cell-, B cell-, NK cell-+, Combined cellular and humoral immune defects with granulomas				
134. #	V272I	TTT GTT- TTT ATT	Un.S	SCID, autosomal recessive, T cell-, B cell-, NK cell-+, Combined cellular and humoral immune defects with granulomas				
135. #	Y277C	TAT-TGT	Un.S	SCID, autosomal recessive, T cell-, B cell-, NK cell-+, Combined cellular and humoral immune defects with granulomas				
136. -	Q278*	CAG-TAG			OS			
137. #	Q282K	CAA-AAA	Un.S	SCID, autosomal recessive, T cell-, B cell-, NK cell-+, Combined cellular and humoral immune defects with granulomas				
138. #-	M285R	ATG-AGG	LP	Recombinase activating gene 2 deficiency, Primary immunodeficiency, HMR, HMR	OS	1 Italian pt who as C41W[7]	1	
139. #	N288D	AAC-GAC	Un.S	SCID, autosomal recessive, T cell-, B cell-, NK cell-+, HMR				
140. #	p. Ser291=	TCT-TCC	LB	SCID, autosomal recessive, T cell-, B cell-, NK cell-+, Combined cellular and humoral immune defects with granulomas				
141. #	E293G	GAG-GGG	B	SCID, autosomal recessive, T cell-, B cell-, NK cell-+, SCID, autosomal recessive, T cell-, B cell-, NK cell-+, Combined cellular and humoral immune defects with granulomas, HMR, not provided, not specified				
142. #	Asp294=	GAC-GAT	LB	NP				
143. #	N295S	AAC-AGC	Un.S	SCID, autosomal recessive, T cell-, B cell-, NK cell-+, Combined cellular and humoral immune defects with granulomas				
144. #	E303G	GAG-GGG	Un.S	SCID, autosomal recessive, T cell-, B cell-, NK cell-+, Combined cellular and humoral immune defects				

					with granulomas			
145.	#	E303D	GAG-GAT	C.P (3Un.S, 1LB)	Combined cellular and humoral immune defects with granulomas, SCID, autosomal recessive, T cell-, B cell-, NK cell-+, SCID, autosomal recessive, T cell-, B cell-, NK cell-+, HMR.			
146.	-	P305A	CCA-GCA			Primary ID		
147.	#	T304I	ACC-ATC	Un.S	HMR			
148.	#	p.Pro305=	CCA-CCG	LB	SCID, autosomal recessive, T cell-, B cell-, NK cell-+, Combined cellular and humoral immune defects with granulomas			
149.	#	D306G	GAT-GAA	Un.S	HMR			
150.	-	W307*	TGG-TGA			SCID, T^- &B^-	1 pt homo[7]	1
151.	#	p.Asp310=	GAC-GAT	LB	SCID, autosomal recessive, T cell-, B cell-, NK cell-+, Combined cellular and humoral immune defects with granulomas			
152.	#	I311T	ATT-ACT	Un.S	SCID, autosomal recessive, T cell-, B cell-, NK cell-+, Combined cellular and humoral immune defects with granulomas			
153.	#	S314I	AGC-ATC	Un.S	SCID, autosomal recessive, T cell-, B cell-, NK cell-+, Combined cellular and humoral immune defects with granulomas			
154.	#	I316M	ATA-ATG	B	SCID, autosomal recessive, T cell-, B cell-, NK cell-+, Combined cellular and humoral immune defects with granulomas			
155.	#	G319*	TTA GGA- TTA TGA	P	Not provided			
156.		V327D	GTT-GAT			SCID	1 Egyptian[6]	1
157.	#	F328S	TTT-TCT	Un.S	SCID, autosomal recessive, T cell-, B cell-, NK cell-+, Combined cellular and humoral immune defects with granulomas			
158.	#	K336E	AAA-GAA	Un.S	Not specified			
159.	#	p.Phe343=	TTC TAT-TTT TAT	LB	SCID, autosomal recessive, T cell-, B cell-, NK cell-+, Combined cellular and humoral immune defects with granulomas			
160.	#	Y346C	TAT-TGT	Un.S	HMR			
161.	#	p.Tyr346=	TAT-TAC	LB	SCID, autosomal recessive, T cell-, B cell-, NK cell-+, Combined cellular and humoral immune defects			

				with granulomas				
162. #	M347I	ATG-ATA	Un.S	SCID, autosomal recessive, T cell-, B cell-, NK cell-+, Combined cellular and humoral immune defects with granulomas				
163. #	p.Glu358=	GAG-GAA	C.P (2Un.S, 1B)	SCID, autosomal recessive, T cell-, B cell-, NK cell-+, HMR				
164. -	Q359G	CAG-GAG			SCID			
165. #	F362L	TTC ACA-TTG ACA	Un.S	SCID, autosomal recessive, T cell-, B cell-, NK cell-+				
166. #	N364K	AAC-AAG	Un.S	SCID, autosomal recessive, T cell-, B cell-, NK cell-+, HMR, Combined cellular and humoral immune defects with granulomas, SCID, autosomal recessive, T cell-, B cell-, NK cell-+				
167. #	Ser365=	AGT-AGC	B/LB	Combined cellular and humoral immune defects with granulomas, SCID, autosomal recessive, T cell-, B cell-, NK cell-+, SCID, autosomal recessive, T cell-, B cell-, NK cell-+, not provided, HMR				
168. #	D374H	GGG GAT-GGG CAT	Un.S	SCID, autosomal recessive, T cell-, B cell-, NK cell+, Combined cellular and humoral immune defects with granulomas				
169. #	p.Pro377=	CCC TTT-CCT TTT	LB	Combined cellular and humoral immune defects with granulomas, SCID, autosomal recessive, T cell-, B cell-, NK cell+				
170. #	D380Y	GAA GAC- GAA TAC	Un.S	SCID, autosomal recessive, T cell-, B cell-, NK cell-+, Combined cellular and humoral immune defects with granulomas				
171. #	p.Asp380=	GAC-GAT	LB	SCID, autosomal recessive, T cell-, B cell-, NK cell-+, Combined cellular and humoral immune defects with granulomas				
172. #	E383K	GAA GAA- GAA AAA	Un.S	SCID, autosomal recessive, T cell-, B cell-, NK cell-+, Combined cellular and humoral immune defects with granulomas				
173. #-	F386L	TTC AGT-TTA AGT	B/LB	Combined cellular and humoral immune defects with granulomas, SCID, autosomal recessive, T cell-, B cell-, NK cell-+, Combined cellular and humoral immune defects with granulomas, SCID, autosomal recessive, T cell-, B cell-, NK cell-+,				

				HMR, not specified, SCID, autosomal recessive, T cell-, B cell-, NK cell-+, Primary immunodeficiency, RAG 2 deficiency, HMR				
174. #	A388E	GCA-GAA	Un.S	SCID, autosomal recessive, T cell-, B cell-, NK cell-+, Combined cellular and humoral immune defects with granulomas				
175. #	p.Ala388=	GCA-GCG	LB	SCID, autosomal recessive, T cell-, B cell-, NK cell-+, Combined cellular and humoral immune defects with granulomas				
176. #	G395A	GGT-GCT	Un.S	SCID, autosomal recessive, T cell-, B cell-, NK cell-+, Combined cellular and humoral immune defects with granulomas				
177. #	D396G	GAT-GGT	Un.S	SCID, autosomal recessive, T cell-, B cell-, NK cell-+, Combined cellular and humoral immune defects with granulomas				
178. #	F399S	TTT-TCT	Un.S	SCID, autosomal recessive, T cell-, B cell-, NK cell-+, Combined cellular and humoral immune defects with granulomas				
179. #	D400H	TTT GAC-TTT CAC	Un.S	SCID, autosomal recessive, T cell-, B cell-, NK cell-+, Combined cellular and humoral immune defects with granulomas, SCID, autosomal recessive, T cell-, B cell-, NK cell-+, HMR				
180. #	p.Asn403=	AAT-AAC	C.P (2Un.S,1LB)	SCID, autosomal recessive, T cell-, B cell-, NK cell-+, HMR				
181. -	E407*	GAT GAA-GAT TAA			Leaky SCID	1 USA homo[15]	1	
182. #	p.Glu407=	GAA-GAG	LB	SCID, autosomal recessive, T cell-, B cell-, NK cell-+, SCID, autosomal recessive, T cell-, B cell-, NK cell-+, Combined cellular and humoral immune defects with granulomas,				
183.	D409E	GAT-GAA			SCID	1 USA homo[15]	1	
184. #	p.Gly414=	GGC-GGA	LB	SCID, autosomal recessive, T cell-, B cell-, NK cell-+, SCID, autosomal recessive, T cell-, B cell-, NK cell-+, Combined cellular and humoral immune defects with granulomas,				
185. #	p.Try415=	TAC-TAT	LB	SCID, autosomal recessive, T cell-, B cell-, NK cell-+,				

				SCID, autosomal recessive, T cell-, B cell-, NK cell-+, Combined cellular and humoral immune defects with granulomas,			
186. #-	W416L	TGG-TTG	C.P (1P, 2LP, 1Un.S)	HMR, Recombinase activating gene 2 deficiency, Primary immunodeficiency, HMR, not provided	OS	1 Italian pt homo[1, 9]	1
187. #	T418I	ACA-ATA	Un.S	SCID, autosomal recessive, T cell-, B cell-, NK cell-+, Combined cellular and humoral immune defects with granulomas			
188.	C419W	TGC TGC-TGG TGC			OS	1 Egyptian homo[6]	1
189. #	C423T	TGT-TAT	Un.S	SCID, autosomal recessive, T cell-, B cell-, NK cell-+, Combined cellular and humoral immune defects with granulomas			
190. #	W430*	TGG-TGA	LP	SCID, autosomal recessive, T cell-, B cell-, NK cell-+, Combined cellular and humoral immune defects with granulomas			
191. -#	P432L	CCA-CTA	Un.S	SCID, autosomal recessive, T cell-, B cell-, NK cell-+, SCID, autosomal recessive, T cell-, B cell-, NK cell-+, Combined cellular and humoral immune defects with granulomas,	SCID	1 Chinese pt[4]	1
192.	Y434H	TAT-CAT			Classical O.S	1 homo Russia,Bashkotostan[24]<br>1 Russia, St.petersburg [24]	2
193. -	Y434S	TAT-TCT			SCID		
194. #	E437K	ACT GAG- ACT AAG	C.P (1LP,2Un.S)	Atypical SCID due to complete RAG1/2 deficiency, Primary immunodeficiency, Recombinase activating gene 2 deficiency, not provided, not specified			
195. #-	K440N	AAA-AAC	L.P	Primary immunodeficiency, HMR, Recombinase activating gene 2 deficiency		1 pt who has P253R [1, 9]	1
196. #	P441T	CCC-ACC	L.P	Immunodeficiency			
197. #	p. Ala442=	GCC-GCT	LB	SCID, autosomal recessive, T cell-, B cell-, NK cell-+, SCID, autosomal recessive, T cell-, B cell-, NK cell-+, Combined cellular and humoral immune defects with granulomas,			

198.	#	M443T	ATG-ACG	LP	SCID, autosomal recessive, T cell-, B cell-, NK cell-+, SCID, autosomal recessive, T cell-, B cell-, NK cell-+, Combined cellular and humoral immune defects with granulomas,				
199.	-	M443I	ATG-ATT			OS			
200.	#-	M443I	ATG-ATT	L.P	Primary immunodeficiency, HMR, Recombinase activating gene 2 deficiency		1 homo pt[1, 2]	1	
201.	#-	I444M	ATC TAC-ATG TAC	C.P (1LP, 1Un.S)	Primary immunodeficiency, HMR, Recombinase activating gene 2 deficiency		1 pt homo[1, 23]	1	
202.	#-	C446W	TGC TCT-TGG TCT	C.P (1LP, 1Un.S)	Primary immunodeficiency, Recombinase activating gene 2 deficiency				
203.	#-	G451A	GGG-GCG	C.P (3P, 1LP, 1Un.S)	Primary immunodeficiency, HMR, Recombinase activating gene 2 deficiency Combined immunodeficiency with skin granulomas, not provided, Common variable immunodeficiency, Combined cellular and humoral immune defects with granulomas, SCID, autosomal recessive, T cell-, B cell-, NK cell-+, Combined cellular and humoral immune defects with granulomas		1 German pt who has T77N[1, 21] 1 USA pt who has G32E [15]	2	
204.	#-	W453R	TGG-AGG	P/L.P	Primary immunodeficiency, HMR, Recombinase activating gene 2 deficiency		1 Netherland homo pt[1, 16]	1	
205.	#	p. Val454=	GTC-GTT	LB	SCID, autosomal recessive, T cell-, B cell-, NK cell-+, SCID, autosomal recessive, T cell-, B cell-, NK cell-+, Combined cellular and humoral immune defects with granulomas,				
206.	#	p. His455=	CAT-CAC	LB	SCID, autosomal recessive, T cell-, B cell-, NK cell-+, SCID, autosomal recessive, T cell-, B cell-, NK cell-+, Combined cellular and humoral immune defects with granulomas,				
207.	#-	A456T	CAT GCT- CAT ACT	L.P	Primary immunodeficiency, HMR, Recombinase activating gene 2 deficiency		1 homo pt[1, 25]	1	
208.	#	A456D	GCT-GAT	Un.S	SCID, autosomal recessive, T cell-, B cell-, NK cell-+,				

					SCID, autosomal recessive, T cell-, B cell-, NK cell-+, Combined cellular and humoral immune defects with granulomas,			
209.	#-	M459L	ATG-CTG	L.P	Primary immunodeficiency, Combined immunodeficiency with skin granulomas, HMR, Recombinase activating gene 2 deficiency, Atypical SCID due to complete RAG1/2 deficiency		1 Russia, St.petersburg who has Y434H[24] 1 USA pt who has G451A[1, 15] 1 homo(2 relatives)[1, 26]	3
210.	#	p.Leu461=	CTG-TTG	L.B	SCID, autosomal recessive, T cell-, B cell-, NK cell-+, SCID, autosomal recessive, T cell-, B cell-, NK cell-+, Combined cellular and humoral immune defects with granulomas,			
211.	#	p. Glu463=	GAA-GAG	LB	SCID, autosomal recessive, T cell-, B cell-, NK cell-+, SCID, autosomal recessive, T cell-, B cell-, NK cell-+, Combined cellular and humoral immune defects with granulomas,			
212.	#	L466F	CTC-TTC	Un.S	Combined cellular and humoral immune defects with granulomas, SCID, autosomal recessive, T cell-, B cell-, NK cell-+, HMR, SCID, autosomal recessive, T cell-, B cell-, NK cell-+			
213.	#	H468R	CAT-CGT	Un.S	SCID, autosomal recessive, T cell-, B cell-, NK cell-+, SCID, autosomal recessive, T cell-, B cell-, NK cell-+, Combined cellular and humoral immune defects with granulomas, HMR			
214.	#	G472R	GCA GGA- GCA AGA	Un.S	SCID, autosomal recessive, T cell-, B cell-, NK cell-+, SCID, autosomal recessive, T cell-, B cell-, NK cell-+, Combined cellular and humoral immune defects with granulomas,			
215.	#-	N474S	AAC-AGC	C.P (1Un.S,1LB)	Primary immunodeficiency, SCID, autosomal recessive, T cell-, B cell-, NK cell-+, Recombinase activating gene 2 deficiency		1 pt homo[1, 7]	1
216.	-	Y476H	TAT-CAT			SCID		
217.	#	p.Tyr477=	TAC-TAT	LB	SCID, autosomal recessive, T cell-, B cell-, NK cell-+, SCID, autosomal recessive, T cell-, B cell-, NK cell-+,			

				Combined cellular and humoral immune defects with granulomas,				
218. -	C478R	TGC-CGC			SCID			
219. #-	C478Y	TGC-TAC	Un.S	SCID, autosomal recessive, T cell-, B cell-, NK cell-+, SCID, autosomal recessive, T cell-, B cell-, NK cell-+, Combined cellular and humoral immune defects with granulomas,		3 pt homo, 1 of them with MFT[1, 7, 12]	3	
220. -	E480*	AAT GAG- AAT TAG				1 homo Israel pt[1, 20]	1	
221. #-	H481P	CAT-CCT	Un.S	Primary immunodeficiency, Recombinase activating gene 2 deficiency		1 Netherland homo[16]	1	
222. #	His481=	CAT-CAC	LB	SCID, autosomal recessive, T cell-, B cell-, NK cell-+, SCID, autosomal recessive, T cell-, B cell-, NK cell-+, Combined cellular and humoral immune defects with granulomas,				
223. #	G472R	GGA-AGA	Un.S	SCID, autosomal recessive, T cell-, B cell-, NK cell-+				
224. #	Leu495=	CTA-CTT CTA-TTA	LB	SCID, autosomal recessive, T cell-, B cell-, NK cell-+				
225. #	p. Leu497=	TTA-TTG	LB	SCID, autosomal recessive, T cell-, B cell-, NK cell-+, SCID, autosomal recessive, T cell-, B cell-, NK cell-+, Combined cellular and humoral immune defects with granulomas,				
226. #-	K498*	AAA-TAA	Un.S	SCID, autosomal recessive, T cell-, B cell-, NK cell-+, SCID, autosomal recessive, T cell-, B cell-, NK cell-+, Combined cellular and humoral immune defects with granulomas,				
227. #	p.Lys499=	AAG-AAA	LB	SCID, autosomal recessive, T cell-, B cell-, NK cell-+, SCID, autosomal recessive, T cell-, B cell-, NK cell-+, Combined cellular and humoral immune defects with granulomas,				
228. #	P501A	CCA-CGA	Un.S	SCID, autosomal recessive, T cell-, B cell-, NK cell-+, SCID, autosomal recessive, T cell-, B cell-, NK cell-+, Combined cellular and humoral immune defects with granulomas,				
229. #-	M502V	ATG-GTG	C.P (7 Un.S, 2B, 2LB)	SCID, autosomal recessive, T cell-, B cell-, NK cell-+, SCID, autosomal recessive, T cell-, B cell-, NK cell-+, Combined cellular and	OS	1 Italian pt who has S160L[1, 9] 1 homo USA[15]	2	

				humoral immune defects with granulomas,HMR				
230.	#	p.Leu505	CTC-CTA	LB	SCID, autosomal recessive, T cell-, B cell-, NK cell-+, SCID, autosomal recessive, T cell-, B cell-, NK cell-+, Combined cellular and humoral immune defects with granulomas,			
231.	#	R506H	CGT-CAT	C.P (2Un.S,1LB)	SCID, autosomal recessive, T cell-, B cell-, NK cell-+,			
232.	#	G509V	GGT-GTT	Un.S				
233.	#	L514F	TTG-TTC	Un.S	SCID, autosomal recessive, T cell-, B cell-, NK cell-+, SCID, autosomal recessive, T cell-, B cell-, NK cell-+, Combined cellular and humoral immune defects with granulomas,			
234.	#	T515N	ACT-AAT	Un.S	SCID, autosomal recessive, T cell-, B cell-, NK cell-+, SCID, autosomal recessive, T cell-, B cell-, NK cell-+, Combined cellular and humoral immune defects with granulomas,			
235.	#	p.Ser520=	TCC TTT-TCT TTT	LB	SCID, autosomal recessive, T cell-, B cell-, NK cell-+, SCID, autosomal recessive, T cell-, B cell-, NK cell-+, Combined cellular and humoral immune defects with granulomas,			
236.	#	L522P	CTT-CCT	Un.S	SCID, autosomal recessive, T cell-, B cell-, NK cell-+, SCID, autosomal recessive, T cell-, B cell-, NK cell-+, Combined cellular and humoral immune defects with granulomas,			
237.	#	p.Leu522	CTT-CTG	LB	SCID, autosomal recessive, T cell-, B cell-, NK cell-+, SCID, autosomal recessive, T cell-, B cell-, NK cell-+, Combined cellular and humoral immune defects with granulomas,			
238.	#	p.Leu525=	TTG-CTG	LB	SCID, autosomal recessive, T cell-, B cell-, NK cell-+, SCID, autosomal recessive, T cell-, B cell-, NK cell-+, Combined cellular and humoral immune defects with granulomas,			
239.	#	D527E	TAG-TAT	Un.S	SCID, autosomal recessive, T cell-, B cell-, NK cell-+, SCID, autosomal recessive, T cell-, B cell-, NK cell-+, Combined cellular and humoral immune defects with granulomas,			

240.							70	

# refers to mutations found in https://www.ncbi.nlm.nih.gov/clinvar?term=179615[MIM]
- refers to mutations found in http://www.hgmd.cf.ac.uk/ac/gene.php?gene=RAG1
#- refers to mutations found in both site
IF neither # nor – is present, refers to mutations found only in a published paper.

References

1. Tirosh, I., et al., *Recombination activity of human recombination-activating gene 2 (RAG2) mutations and correlation with clinical phenotype.* J Allergy Clin Immunol, 2019. **143**(2): p. 726-735.
2. Asai, E., et al., *Analysis of mutations and recombination activity in RAG-deficient patients.* Clin Immunol, 2011. **138**(2): p. 172-7.
3. Luk, A.D.W., et al., *Family History of Early Infant Death Correlates with Earlier Age at Diagnosis But Not Shorter Time to Diagnosis for SCID.* Front Immunol, 2017. **8**: p. 808.
4. Bai, X., et al., *Clinical, immunologic, and genetic characteristics of RAG mutations in 15 Chinese patients with SCID and Omenn syndrome.* Immunol Res, 2016. **64**(2): p. 497-507.
5. Meshaal, S., et al., *Mutations in Recombination Activating Gene 1 and 2 in patients with SCID disorders in Egypt.* Clin Immunol, 2015. **158**(2): p. 167-73.
6. Meshaal, S.S., et al., *Phenotypical heterogeneity in RAG-deficient patients from a highly consanguineous population.* Clin Exp Immunol, 2019. **195**(2): p. 202-212.
7. Villa, A., et al., *V(D)J recombination defects in lymphocytes due to RAG mutations: severe immunodeficiency with a spectrum of clinical presentations.* Blood, 2001. **97**(1): p. 81-8.
8. Tabori, U., et al., *Detection of RAG mutations and prenatal diagnosis in families presenting with either T-B- SCID or Omenn's syndrome.* Clin Genet, 2004. **65**(4): p. 322-6.
9. Sobacchi, C., et al., *RAG-dependent primary immunodeficiencies.* Hum Mutat, 2006. **27**(12): p. 1174-84.
10. Safaei, S., et al., *IL7R and RAG1/2 genes mutations/polymorphisms in patients with SCID.* Iran J Allergy Asthma Immunol, 2011. **10**(2): p. 129-32.
11. Signorini, S., et al., *Intrathymic restriction and peripheral expansion of the T-cell repertoire in Omenn syndrome.* Blood, 1999. **94**(10): p. 3468-78.
12. Schwarz, K., et al., *RAG mutations in human B cell- SCID.* Science, 1996. **274**(5284): p. 97-9.
13. Corneo, B., et al., *Identical mutations in RAG1 or RAG2 genes leading to defective V(D)J recombinase activity can cause either T-B-severe combined immune deficiency or Omenn syndrome.* Blood, 2001. **97**(9): p. 2772-6.
14. Fazlollahi, M.R., et al., *Clinical, Laboratory, and Molecular Findings for 63 Patients With SCID: A Decade s Experience.* J Investig Allergol Clin Immunol, 2017. **27**(5): p. 299-304.
15. Walter, J.E., et al., *Broad-spectrum antibodies against self-antigens and cytokines in RAG deficiency.* J Clin Invest, 2015. **125**(11): p. 4135-48.
16. Noordzij, J.G., et al., *The immunophenotypic and immunogenotypic B-cell differentiation arrest in bone marrow of RAG-deficient SCID patients corresponds to residual recombination activities of mutated RAG proteins.* Blood, 2002. **100**(6): p. 2145-52.
17. Lev, A., et al., *Characterizing T cells in SCID patients presenting with reactive or residual T lymphocytes.* Clin Dev Immunol, 2012. **2012**: p. 261470.
18. Corneo, B., et al., *Three-dimensional clustering of human RAG2 gene mutations in severe combined immune deficiency.* J Biol Chem, 2000. **275**(17): p. 12672-5.
19. Villa, A., et al., *Partial V(D)J recombination activity leads to Omenn syndrome.* Cell, 1998. **93**(5): p. 885-96.
20. Dalal, I., et al., *Novel mutations in RAG1/2 and ADA genes in Israeli patients presenting with T-B-SCID or Omenn syndrome.* Clin Immunol, 2011. **140**(3): p. 284-90.

21. Schuetz, C., et al., *An immunodeficiency disease with RAG mutations and granulomas.* N Engl J Med, 2008. **358**(19): p. 2030-8.
22. Gomez, C.A., et al., *Mutations in conserved regions of the predicted RAG2 kelch repeats block initiation of V(D)J recombination and result in primary immunodeficiencies.* Mol Cell Biol, 2000. **20**(15): p. 5653-64.
23. Alsmadi, O., et al., *Molecular analysis of T-B-NK+ SCID and Omenn syndrome cases in Saudi Arabia.* BMC Med Genet, 2009. **10**: p. 116.
24. Sharapova, S.O., et al., *Molecular Characteristics, Clinical and Immunologic Manifestations of 11 Children with Omenn Syndrome in East Slavs (Russia, Belarus, Ukraine).* J Clin Immunol, 2016. **36**(1): p. 46-55.
25. Ktiouet, S., et al., *Omenn syndrome due to mutation of the RAG2 gene.* J Eur Acad Dermatol Venereol, 2009. **23**(12): p. 1449-51.
26. Chou, J., et al., *A novel homozygous mutation in recombination activating gene 2 in 2 relatives with different clinical phenotypes: Omenn syndrome and hyper-IgM syndrome.* J Allergy Clin Immunol, 2012. **130**(6): p. 1414-6.

Table S3. The number of columns containing ancestral CpG dinucleotides in both RAG1 and RAG2 alignments.

	**RAG1**	**RAG2**
Columns with conserved CGs	23	8
Columns with mutated CG to TG	95	31
Columns with mutated CG to CA	56	26
Columns with mutated CG to CA/TG ^	15	1
Sum	189	67
The expected original CpG density (before mutation) *	6.03%	4.2%

^ means that columns in this case contain almost equal number of CA and TG.
* Indicates that it was calculated by dividing the whole number of ancestral CpGs (the sum of mutated and non-mutated) for RAG1 or RAG2 into their whole length 3130bp or1584 bp, respectively, then the results were multiplied by 100.
Only columns containing at least 3 CGs dinucleotides were considered in the table.
